# Determinants of child body weight categorization in parents and health care professionals: An experimental study

**DOI:** 10.1111/bjhp.12765

**Published:** 2024-11-14

**Authors:** Elizabeth H. Evans, Bethany J. Ridley, Piers L. Cornelissen, Robin S. S. Kramer, Vera Araújo‐Soares, Martin J. Tovée

**Affiliations:** ^1^ Department of Psychology Durham University Durham UK; ^2^ Department of Psychology Northumbria University Newcastle upon Tyne UK; ^3^ School of Psychology University of Lincoln Lincoln UK; ^4^ Center for Preventive Medicine and Digital Health Medical Faculty Mannheim, Heidelberg University Mannheim Germany

**Keywords:** BMI categories, childhood weight, healthcare professionals, overweight, parents

## Abstract

**Objectives:**

Parents infrequently recognize childhood overweight/obesity and healthcare professionals (HCPs) also struggle to visually identify it, potentially limiting the offer and uptake of weight management support. This study examined perceptual and attitudinal/cognitive determinants of child weight judgements amongst parents and HCPs to identify targets for intervention.

**Design:**

We used a mixed experimental design with parents and HCPs as the between‐participants factor. Stimulus gender, age and BMI centile were the within‐participant repeated measures factors.

**Methods:**

One hundred and fifty‐six HCPs and 249 parents of children aged 4–5 or 10–11 years viewed simulated child images. They estimated their relative size and categorized the weight status of each figure. Stimuli were photo‐realistic figural scales based on 3D‐scans of 4‐ to 5‐ and 10‐ to 11‐year‐old children varying in adiposity. Participants also reported their beliefs about causes, controllability and categorization of child weight.

**Results:**

Both groups accurately estimated the figures' relative size. However, categorization of higher weight figures was poor, demonstrating a mismatch between perceptual judgements of size and categorization of weight status. Lower levels of comfort with assigning ‘overweight’ categorizations to children, and a stronger belief that weight was controllable by the child/parent, predicted less accurate weight status categorizations.

**Conclusions:**

Parental and HCP misperceptions when categorizing children's higher weight are related to attitudinal/cognitive factors, including reluctance to label a child's weight status as overweight and beliefs about whether a child's weight can be controlled by them or their family.

## INTRODUCTION

Childhood overweight and obesity is a significant global public health challenge, affecting an increasing proportion of youth (Abarca‐Gómez et al., 2017; Sahoo et al., 2015). Excess weight in childhood predicts negative physical and psychosocial sequelae over the lifespan, as weight status typically persists over time (Geserick et al., 2018). Consequences of childhood overweight and obesity include some cancers, cardiovascular disease, type 2 diabetes (Wang et al., 2011), depression, anxiety and low self‐esteem (Rankin et al., 2016). In the UK, the body mass index (BMI) of 26% of children aged 4–5 years is in the overweight and obese range, and this increases to 36% of 10‐ to 11‐year‐olds (NHS Digital, 2023), as defined by the UK90 growth reference data (Wright et al., 2002). Fortunately, family and school‐based behavioural interventions can reduce childhood overweight and obesity (Berge & Everts, 2011; Ickes et al., 2014) and there are behaviour change techniques that can be used to support families with children aged 4–11 years (Wehling et al., 2020). However, intervention uptake depends at least partially on parent, caregiver or healthcare professionals' (HCPs') recognition of the need for weight management support (Davidson & Vidgen, 2017; Finne et al., 2009; Kelleher et al., 2017; Perez et al., 2015). Parents systematically underestimate their child's weight status (Lundahl et al., 2014) and HCPs may similarly struggle to visually identify overweight and obesity in children (Benson et al., 2009; Perrin et al., 2012). It is unclear why parents and HCPs miscategorize child weight, so research to identify determinants of child weight status perception is needed to facilitate effective weight‐related decision‐making for both groups. The current study investigated for the first time whether perceptual and/or attitudinal factors predict child weight miscategorization in parents and HCPs. This has the potential to highlight targets for future intervention development efforts (Araújo‐Soares et al., 2018) to improve the identification of children who would benefit from weight management support.

### Misperception of child body weight: Extant research and theory

It is unclear why parental weight miscategorization of weight status occurs: research has implicated factors including lower levels of parental education and health literacy, higher parental BMI and child/parent gender and age, yet the findings are conflicting (Alshahrani et al., 2021; Garrett‐Wright, 2011). The media represents in critical and stigmatizing terms parents' tendency to underestimate the weight status of their higher‐weight child(ren), portraying them as ignorant and unwilling to accept reality (e.g., Elsom, 2019). By contrast, visual normalization theory proposes that children's higher weight goes under‐detected because it is judged relative to body size norms, and larger bodies are becoming more common (Robinson, 2017). It is argued that this has triggered a long‐term recalibration and upward shift in the perceptual threshold for the detection of a child being overweight (Jones et al., 2011; Robinson, 2017).

Additionally, two perceptual factors explain errors in weight and size perception for adult figures: contraction bias and Weber's law (Cornelissen et al., 2016). Although seldom considered in the parental weight recognition literature, Evans et al. (2023) recently demonstrated that these perceptual factors likely also explain weight perception errors for child figures. Contraction bias occurs because we use an internal ‘reference’ body to make size estimations (based on all the bodies ever seen). Our judgements are most accurate when a body is similarly sized to this reference (Cornelissen et al., 2013; Poulton, 1989) and least accurate when a body is very dissimilar, resulting in size overestimation of a smaller‐than‐reference body and underestimation of a larger‐than‐reference body. Weber's law (Gescheider, 1997) also specifically explains under‐recognition of higher weight in children: it states that size differences between bodies are more easily noticed at the lower end of the BMI scale than the upper end. This is because the just noticeable difference (JND) between two objects (minimum size‐change necessary to detect a difference) is a fixed percentage of the size of the original object. These two visual biases lead us to expect lower accuracy in weight judgements with centile‐based stimuli for higher (and lower) than average BMI (Cornelissen et al., 2013, 2016).

Cognitive /attitudinal explanations for parental patterns of weight status categorization have received still less research attention, although significant amounts of health psychology research link behaviour and behaviour change to social‐cognitive predictors (Stacey et al., 2015; Young et al., 2014). These are understood as mechanisms of action (MoA) that can be targeted with key behaviour change techniques to support change (Carey et al., 2019). Such explanations hinge on the idea that parents assign incorrect weight categories because they either misapply or actively avoid them. Terms such as ‘overweight’ or ‘obese’ are widely considered stigmatizing (Puhl, 2020) and a small body of evidence indicates that parents hesitate to apply them to their children (Gainsbury & Dowling, 2018). This could extend to a reluctance to apply the terms to children more generally (Evans et al., 2023). Consequently, parents may be able to accurately assess the relative size of children's bodies but may be reluctant to assign a potentially negative weight descriptor. This may partially or fully explain the patterns of miscategorization documented in the existing research (Lundahl et al., 2014; Parry et al., 2008; Queally et al., 2018) but surprisingly no previous research with parents has examined the relationship between miscategorization and participants' beliefs about child weight categories or comfort with assigning such descriptors. Another hitherto unexplored correlate of weight status categorization is parents' own attitudes to eating, weight and body size. Beliefs about one's own diet and body may feasibly influence the way adults perceive, classify and feel about child weight (e.g., Blanchet et al., 2019).

### Healthcare professional misperception of child weight

HCPs show similar patterns of weight category assignment (e.g., Benson et al., 2009). Even those working regularly with children underestimate the weight status of higher‐weight figures and overestimate lower‐weight figures (e.g., King et al., 2015). HCP detection of child weight is important since they are well placed to signpost to nutritional and/or paediatric weight management support (Lai et al., 2022). Even when they recognize higher weight, many HCPs struggle to discuss weight with families (MacTavish et al., 2020) due to low confidence, lack of understanding and feelings of intimidation (Bradbury et al., 2018). Identical patterns of underestimation are seen when HCPs make judgements of adults. In a sample of trainees and qualified general practitioners, it was found that adults with overweight/obesity were perceived as having a lower BMI and lower weight status. Such miscategorizations were associated with a lower reported intention to discuss weight management with the patient (Robinson et al., 2014). HCPs have called for support and resources to enhance their skill and confidence in weight‐related communications, and a vital part of this is facilitating effective detection of child weights that may benefit from medical or behavioural intervention (MacTavish et al., 2020). Moreover, HCP miscategorization of child weight is potentially driven by similar perceptual, attitudinal/cognitive determinants to parents. Therefore, the current study assessed determinants of HCP weight miscategorization, using the same procedure as that used with parents.

### Study rationale

Participants were presented photo‐realistic figural scales based on 3D‐scans of 4–5‐ and 10‐11‐year‐old children. First, they rated the size of each figure within an image set varying from very low to very high BMI, using a continuous (non‐categorical) scale. This allowed us to establish whether they could effectively and *directionally* distinguish between figures depicting different BMI centiles. Secondly, participants labelled each figure with one of four possible weight categories to establish whether they could assign the correct descriptor. We thus assessed two potential causes of miscategorization: (a) participants' perceptual difficulty in *detecting size differences* between the figures in Task 1; and/or (b) participants' failure to *correctly assign categorical descriptors* in Task 2, for attitudinal /cognitive reasons. To ascertain whether attitudes towards child weight explained effects, participants completed questions to assess their beliefs about child weight and their level of comfort with assigning higher weight descriptors. Participants also completed questionnaire measures of dietary restraint, self‐esteem and body appreciation to ascertain whether individual differences in body‐ and eating‐related psychological variables were associated with patterns of miscategorization.

Participants were HCPS or parents of children aged 4–5 or 10–11 years old. Previous research has never compared the patterns of weight misperception of parents to that of HCPs, another group which often has professional involvement with children and their weight. An important caveat to this objective is that many HCPs are also parents or grandparents, so some overlap between our two participant groups was expected. To account for this, a sensitivity analysis was planned.

### Hypotheses

We expected that both HCPs and parents would accurately detect size differences between stimuli (task 1) but miscategorize child stimuli above and below the healthy weight range (task 2). Specifically, we expected that both underweight and (very) overweight stimuli would be incorrectly categorized as healthy weight, coherent with existing evidence (e.g., Benson et al., 2009; Queally et al., 2018). Finally, we hypothesized that the dissociation between size estimation and weight categorization would be at least partially predicted by attitudinal variables such as weight‐related beliefs (e.g., Evans et al., 2023), so we planned and preregistered an exploratory analysis examining attitudinal covariates of response patterns (see https://osf.io/hzunv/).

## METHODS

### Ethics

Ethical approval was granted by the Psychology Department ethical committee at Northumbria University, Newcastle University's Faculty of Medical Sciences Research Ethics Committee and Durham University Psychology Department Research Ethics Committee.

### Participants

We recruited 156 HCPs—107 from the Prolific online participant pool and 49 via word of mouth. Eligible participants currently worked or studied in a healthcare setting (providing their full job title to confirm HCP status). Individuals in administrative roles (e.g., medical secretary) were excluded, but allied health professionals (e.g., physiotherapists) were included. Additionally, we recruited 249 parents from the Prolific online participant pool amongst whom were 132 parents of children aged 4–5 and 117 parents of children aged 10–11 years. Participants were eligible if they were parents/carers to children aged 4–5 and/or 10–11 years on the date of study completion and were not HCPs. Parents with children of these ages were selected because the National Child Measurement Programme (NCMP) measures children aged 4–5 and 10–11 years as part of its national paediatric weight surveillance programme in England (Parkinson et al., 2015). Across both groups, participants recruited from Prolific received modest remuneration, whilst HCPs recruited via word of mouth received no compensation.

### Stimulus generation

Jones et al. (2018) utilized 3D surface body scanning technology and captured accurate representations of 388 children aged 4–5 and 10–11 years old to create 3D Body Image Scales and the bodies in these scales are directly based on the average 3D shape possessed by children of a particular age, gender and BMI. In a previous behavioural validation study, participants were able to judge the relative size of each body and sort them in ascending order by BMI (Ridley et al., 2024), and the scales have been effectively used as a visual aid to communicate about child weight (Jones et al., 2023; Tommerup et al., 2021). These child age groups were chosen because the National Child Measurement Programme (NCMP) measures the height and weight of children in English schools at these ages as part of its national paediatric weight surveillance programme (Parkinson et al., 2015).

**FIGURE 1:**
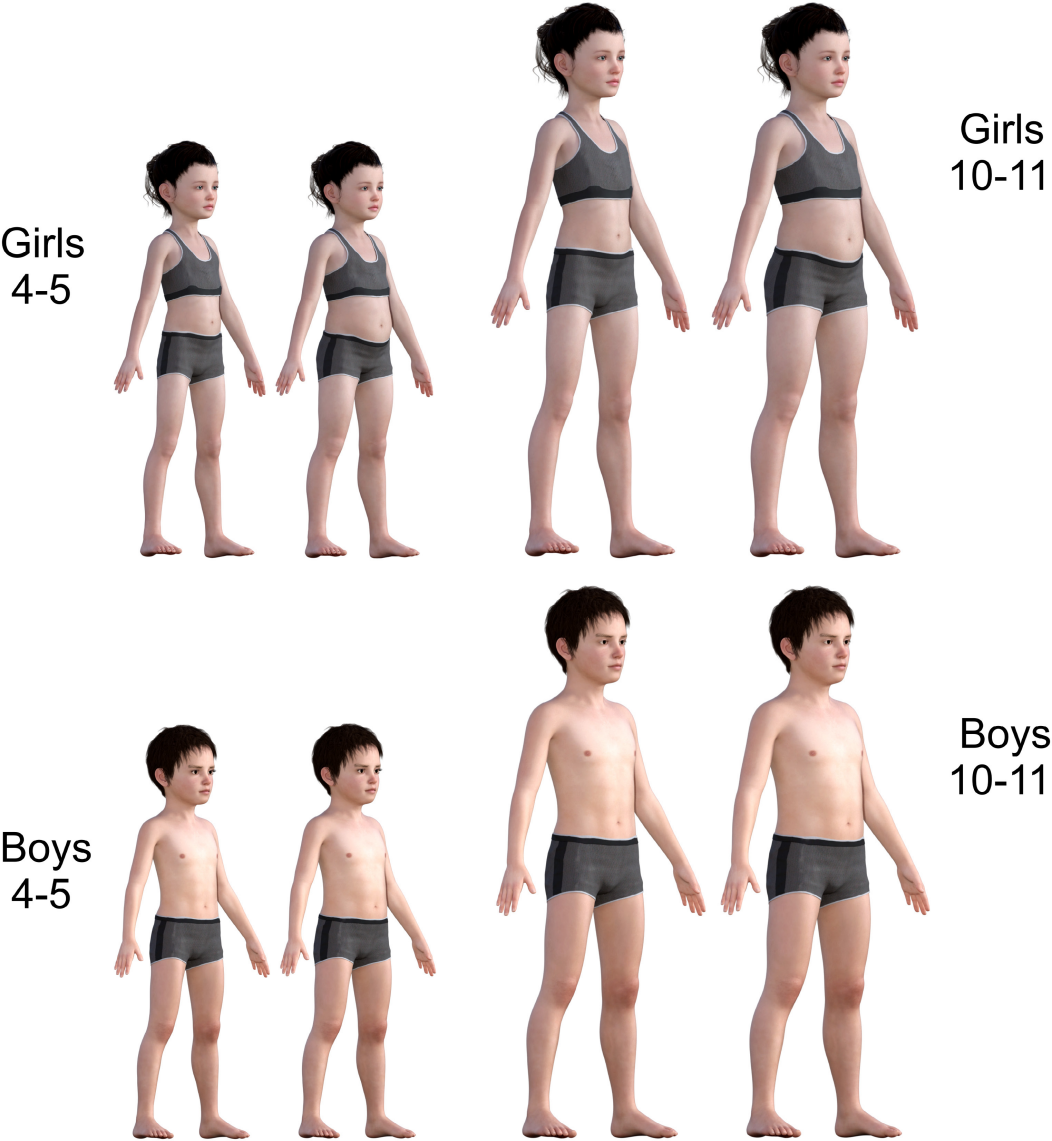
Example stimuli showing older and younger boys and girls at the 25th and 75th BMI centiles.

For each set of images (one boys set and one girls set at each age), seven weight categories were represented: underweight (2nd centile); lower‐healthy weight (25th centile); mid‐healthy weight (50th centile); upper‐healthy weight (75th centile); overweight (91st centile); lower‐very overweight (98th centile); and upper‐very overweight (99.6th centile) (for examples see Figure 1). The categories represented were guided by the NCMP's cut‐offs and labelling terminology (Public Health England, 2021).

### Procedure

The survey and information/consent documents were hosted online via Qualtrics. Participants first read an online information sheet describing the study purpose and methods, right to withdraw and data storage and handling. Participants then provided informed consent. Before proceeding with the demographic information, completing psychometric questionnaires and providing weight ratings of each individual image, participants were first asked to confirm that they were using either a laptop or desktop, rather than a mobile phone or tablet; if one of the latter two was chosen, the survey terminated. This exclusion was a precaution designed to prevent participants viewing each image on a small screen which might make it difficult to clearly perceive image details. If participants could not clearly see the image, then the study would not be a good test of their ability to judge its (relative) weight status. All participants then reported their age, gender (man, woman, prefer not to say, prefer to self‐describe), weight (in stones and pounds, kilograms, or pounds) and height (in feet and inches, metres, or centimetres).

Participants in the HCP sample specified the field in which they worked (or studied) for most of the time (e.g., general practice, paediatric medicine) and provided their job title. They reported their employment status (e.g., full time) and then reported whether their role involved frequent work with children (e.g., most days or daily) and in which country they worked. They indicated whether they had any children under the age of 18, how many, and their age(s) and gender(s).

At the start of the parents' survey, participants completed a pre‐screen question to confirm that they had a child of the required age. If the participant's response was inconsistent with details stored in their Prolific profile (i.e., reported a priori at the point of registering with Prolific), their survey participation was ended. Parents reported their occupation and whether they worked (or studied) in healthcare, and if so, were excluded. Non‐HCP parents also described their job role and indicated whether they worked with children. Finally, they confirmed how many children under 18 they had, as well as their age(s) and gender(s).

### Beliefs about childhood obesity and psychometric questionnaires

Next, respondents used three separate visual analogue scales to assess the extent to which they believed genetics, overeating and lack of physical activity caused child obesity (0 = ‘not at all’, 100 = ‘completely’). They also used two additional visual analogue scales to rate (i) how controllable they believed child obesity to be by children or their family, and (ii) how comfortable they felt in assigning the descriptors ‘overweight’ and ‘very overweight’ to children. These items have been used in previous studies to assess participant attitudes to child weight (Evans et al., 2023; Parkinson et al., 2015) and, individually, show convergent validity with existing measures of attitudes towards higher weight such as the Anti‐Fat Attitudes Scale (Evans et al., 2023). The item asking about comfort in assigning the ‘(very) overweight’ descriptors was developed with groups of parents and healthcare professionals to ensure that it appropriately captured the experience of using these labels for children's body size.

Participants then completed three psychometric questionnaires, which included the Body Appreciation Scale‐2 (BAS‐2; Tylka & Wood‐Barcalow, 2015), the Rosenberg Self Esteem Scale (RSE; Rosenberg, 1965) and the 6‐item Three Factor Eating Questionnaire‐R18 Restraint subscale (TFEQ‐R18; Karlsson et al., 2000).

The BAS‐2 is a 10‐item questionnaire that aims to assess assesses individuals' acceptance of, favourable opinions towards, and respect for their bodies (Tylka & Wood‐Barcalow, 2015). Each item of the questionnaire is measured on a 5‐point Likert scale from never (1) to always (5) and an example item is ‘I am attentive towards my body's needs’. The BAS‐2 has been shown to withhold a unidimensional structure across sex and sample type, internally consistent and has evidenced construct validity (convergent, discriminant and incremental) (Tylka & Wood‐Barcalow, 2015). The Cronbach's alpha of the BAS‐2 in this sample was .957 and .961 for the HCP and parent sample respectively.

The Rosenberg Self Esteem Scale (RSE) is a 10‐item questionnaire that consists of statements that cover a range of positive and negative feelings about oneself, half of which are reverse‐keyed. Respondents indicate their level of endorsement of each statement on a 4‐point Likert scale from strongly agree (0) to strongly disagree (3). An example statement is ‘I take a positive attitude toward myself.’. Total scores on this measure range from 0 to 30, with higher scores indicating higher self‐esteem. The RSE has demonstrated good psychometric properties, including good predictive validity, good internal consistency and good test–retest reliability (Schmitt & Allik, 2005; Torrey et al., 2000). In the HCP and parents' samples, Cronbach's alpha for the RSE were .890 and .936, respectively.

The dietary restraint subscale from the Three Factor Eating Questionnaire‐R18 (Karlsson et al., 2000) was used to assess participants' reported attempts to restrict or reduce their dietary intake to control body weight. Respondents indicate their level of endorsement of six statements using a scale of (1)–(4) for five items and a scale of (1)–(8) for the sixth. An example statement is ‘I do not eat some foods because they make me fat’. The dietary restraint subscale shows good to acceptable internal consistency, convergent and divergent validity (Karlsson et al., 2000). In the current sample, Cronbach's alpha for all six TFEQ items was .797 and .782 in the HCP and parent samples, respectively.

### Body weight perception tasks

Finally, participants completed the body weight perception tasks. HCPs completed the tasks for all sets of all the child stimuli, however, parents only judged the stimulus sets that aligned the age of the eldest child (i.e. only 4‐ to 5‐ or 10‐ to 11‐year‐olds). Each child figure appeared individually on the screen during these tasks. First, participants categorized each individual figure's weight status using the NCMP categories (Underweight, Healthy Weight, Overweight, Extremely Overweight). Second, participants indicated where the weight of each individual figure lay between extremely underweight to extremely overweight using a visual analogue scale (0–100). Stimulus presentation order was randomized across participants, for each of the 28 figures shown (7 each for younger girls, older girls, younger boys and older boys). Thus, in this study, each image was judged individually for its size and weight status category. This is a more challenging test of size estimation than making these judgements when the other bodies within a set are on view, as the participant does not have the other bodies as reference but must make each judgement in isolation. It also might be argued that this is a more realistic approximation of the body size judgements our participants are making in normal life, as they are unlikely to make judgements about a child's size when viewing them as part of an array of children of the same age and gender.

For the VAS weight scores, the size of each body was estimated by assigning it a score between 0 to 100. Therefore, for the 7 body size levels (i.e., BMI centiles: 2, 25, 50, 75, 91, 98, 99.6), we could compute mean VAS weight scores for each level and ask whether successive VAS weight scores from one BMI centile to the next were significantly different. If so, this was evidence of clear discrimination between BMI centiles. For the categorization data, we calculated the probabilities of correct assignment to weight category.

In this study the individual images were not judged as part of a simultaneously presented array of images. However, in a previous validation study for these sets of body scales, control participants not only judged the size of the bodies in the same VAS weight task as used here but also viewed all the bodies in an array and sorted them in ascending order, a task which they were able to successfully complete (Ridley et al., 2024).

### Sample size calculation

This study examined whether there is a mismatch between participants' performance at categorizing child weight and detecting whether a given child is heavier or lighter than another, irrespective of weight category. To derive a sample size estimate, a sample of 31 adults completed a visual analogue scale (VAS) detection task. They viewed images corresponding to the 2nd, 25th, 50th, 75th, 91st, 98th and 99.6th BMI centiles and rated each image's size using a VAS (very underweight to very overweight). We determined the smallest detectable difference in VAS weight scores between successive centiles and used the mean difference score and the standard deviation of these differences to calculate Cohen's *d*_z_. We then used G*Power (Faul et al., 2009) to estimate the sample size required to detect this difference with *α* = .01, and power (1 − β = .95) using a *t*‐test for matched pairs. We argue that this is a stringent criterion to demonstrate a dissociation, and if discrimination/categorization performance is poor, we can be confident that detection has been robustly demonstrated. This required a sample size of at least 109 per group. We recruited for both groups until we exceeded this sample size with complete responses.

### Data quality checks

Although Prolific has been shown to provide some of the highest data quality for online human‐participants research (Douglas et al., 2023), attention checks were included in the online surveys. An attention check item was added to the RSE that asked participants to ‘please select “agree” to demonstrate that you are paying attention’. A second attention check was added at the end of the survey, which required respondents to use a visual analogue scale (0–100) to ‘please position this slider at 90’. Another indicator of data quality is how long participants spend taking the survey, as responses that are returned too quickly can indicate worse data quality (Douglas et al., 2023). We therefore reviewed the response times for all participants to ensure that no responses were returned too quickly, defined as more than three standard deviations below the mean completion time.

### Statistical analysis

To determine whether participants could discriminate between different body sizes, we grouped parents of 4‐ to 5‐year‐olds and 10‐ to 11‐years‐olds together. In addition, for the VAS weight data, we wanted to focus on two constraints: (a) preserving the relative ordering of individual participant responses, while (b) retaining the ability to discriminate the quantitative differences in VAS weight scores assigned to successive BMI centiles, especially across stimulus sex and age. Had we simply used rank scoring to deal with the former constraint, this would have eliminated our ability to consider the second constraint. However, to admit the second constraint opens the possibility of additional, unwanted variation, due to range equalizing and centring biases (Poulton, 1989). In other words, different participants might use different ranges for the response scale (e.g., 30–70 vs. 15–85) and/or different locations for the range they used (e.g., 5–55 vs. 25–75). If so, such additional variation would potentially obscure the relative discriminability between successive BMI centiles across participants. Therefore, as a compromise, we chose to normalize the VAS weight data per participant. To do this we computed the range of VAS weight responses for each participant across the two stimulus sexes and ages. We then subtracted the smallest VAS weight response from all responses. Finally, we divided these corrected values by the range and multiplied by 100 to render the normalized VAS weight scores (see, e.g., Geng et al., 2012; Patcas et al., 2019).

We used PROC MIXED (SAS v9.4) to build linear mixed effects models with normalized VAS weight score as the outcome variable. As fixed effects we tested: group (HCPs and parents), BMI centile and stimulus type (younger girls, older girls, younger boys and older boys). As covariates we tested: (a) participant age, gender and BMI, (b) participant ratings of genetic, overeating and physical activity weight causality attributions, (c) participant ratings for comfort assigning higher weight descriptors to children and controllability of child weight by children/family and (d) participant responses to the psychometric questionnaires (BAS, RSE, TFEQ).

To assess participants' categorization accuracy, we computed generalized linear mixed models (GLMMs), fitted with PROC GLIMMIX (SAS v9.4). Dichotomous match/mismatch was regressed upon a series of pre‐specified predictors. As fixed effects, we tested: stimulus weight category (i.e., 1–4), participant group (i.e., HCPs and parents) and stimulus type (i.e., younger girls, older girls, younger boys and older boys). In addition, we tested the same covariates as for the VAS weight data analysis, above.

## RESULTS

The study was pre‐registered at https://doi.org/10.17605/OSF.IO/EWNQ7 and the data are available at https://osf.io/hzunv/.

### Univariate statistics

We obtained complete datasets for 156 HCPs (80.8% women, 18.6% men, .6% non‐binary), 132 parents of 4–5‐year‐old children (47% women, 53% men) and 115 parents of 10–11‐year‐old children (47.8% women, 49.5% men, 1.7% non‐binary), giving a total of 402 participants. One hundred and seven of the HCPs and all 247 parents were from the Prolific participant pool. It proved challenging to recruit HCPs who did not have children under the age of 18 and such a sample would be unrepresentative of HCPs more broadly. As a result, 89 had children aged <18 years. Table 1 shows their group characteristics.

**TABLE 1:** Characteristics of participants.

Observer	HCPs (women = 126, men = 29)		Parents 4‐ to 5‐year‐olds (women = 62, men = 70)		Parents 10‐ to 11‐year‐olds (women = 55, men = 60)	
	*M*	*SD*	*M*	*SD*	*M*	*SD*
Women						
Age (yrs)	41.71	10.18	35.84	5.18	38.11	6.040
BMI (kg/m^2^)	27.65	7.46	26.53	7.64	27.88	6.86
Genetic attribution	38.36	24.49	41.21	23.12	42.04	24.28
Overeating attribution	74.30	17.42	78.53	15.96	77.64	14.64
Activity attribution	73.78	18.47	78.37	16.67	74.71	21.10
Comfort	48.38	30.31	44.32	28.75	45.33	30.53
Controllability beliefs	74.08	16.59	75.74	13.72	80.07	11.34
Body appreciation	31.98	8.87	31.29	8.59	31.85	9.60
Self‐esteem	14.73	3.47	14.31	4.10	13.49	4.70
Dietary restraint	14.72	4.48	13.45	3.71	14.58	4.30
Men						
Age (yrs)	41.86	12.10	36.81	6.65	43.18	7.050
BMI (kg/m^2^)	26.52	4.65	27.74	5.46	28.06	7.00
Genetic attribution	38.28	24.29	38.89	21.90	37.00	21.81
Overeating attribution	69.38	20.32	79.57	16.21	79.30	11.50
Activity attribution	64.48	23.84	74.57	19.69	78.43	14.31
Comfort	64.17	25.46	57.93	30.72	62.15	31.35
Controllability beliefs	76.59	14.98	84.09	12.15	79.55	15.71
Body appreciation	33.10	7.36	33.33	9.13	32.78	8.01
Self‐esteem	13.34	5.92	12.71	4.45	12.43	3.87
Dietary restraint	13.83	3.26	14.28	3.96	15.03	3.87

*Note*: Genetic/overeating/activity attribution: causal beliefs about child weight; Comfort: comfort assigning higher weight descriptors to children; Controllability: belief that child weight can be controlled by the child and/or their family; Body appreciation: Body Appreciation Scale‐2; Self‐esteem: Rosenberg Self‐esteem Scale; Dietary restraint: Three Factor Eating Questionnaire‐18R Restraint subscale.

Abbreviation: BMI, body mass index.

### Could participants discriminate between different body sizes, and which variables predicted their ability to do so?

In the linear mixed effects model of normalized VAS weight scores, Type III tests of fixed effects showed a significant effect of BMI centile (*F*_(1,6744)_ = 657.12, *p* < .0001). This indicates that normalized VAS scores increased systematically as a function of BMI centile. A fixed effect of stimulus type (*F*_(3,2134)_ = 53.56, *p* < .0001) is attributable to the higher normalized VAS scores for girls (older *M* = 57.3, younger = 57.3) than for boys (older *M* = 55.9, younger = 51.5). To show the directions of these effects, and to see the full model parameters, please refer to Tables S1 and S2 of the Supporting Information (Tables S3 and S4 show the model parameters for an equivalent analysis of the raw, unnormalized VAS weight scores). We found a significant participant group × BMI centile interaction (*F*_(6,6743)_ = 5.71, *p* = .01) due to the narrower range of normalized‐VAS scores for HCPs (16.0–80.4) than for parents (13.7–84.8) across BMI centiles. In addition, we found a significant stimulus type × BMI centile interaction (*F*_(18,6743)_ = 38.39, *p* < .0001) due to the narrower range of normalized VAS scores for boys (older = 24.7–64.5, younger = 16.2–76.7) than for girls (older = 8.0–83.4, younger = 10.5–85.4) across BMI centiles. There was also a significant three‐way interaction, participant group × BMI centile × stimulus type (*F*_(21,5150)_ = 1.75, *p* = .02). This was due to the fact that the wider range of normalized VAS scores for girls, across the BMI centiles, is larger for parents than for HCPs.

**FIGURE 2:**
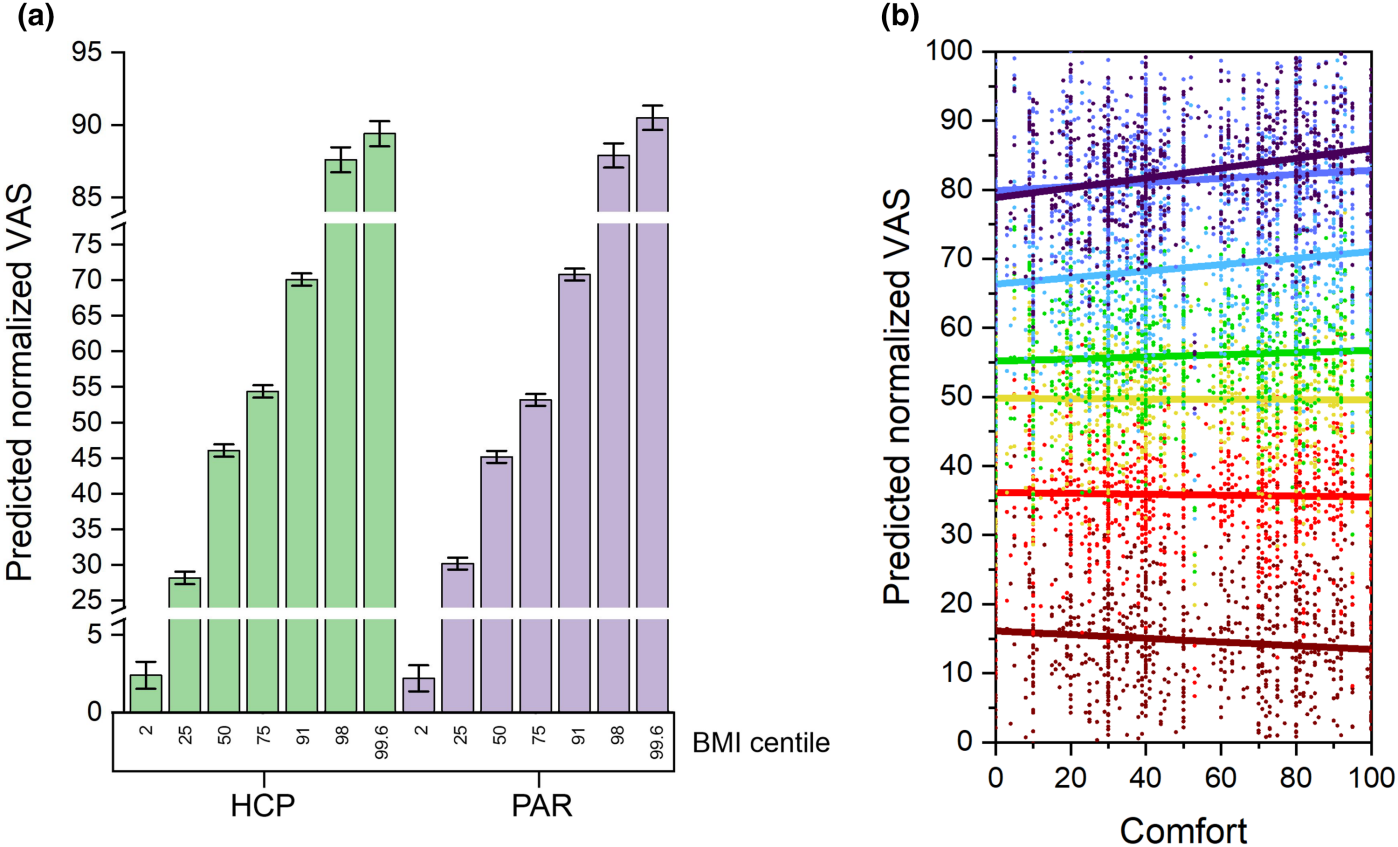
(a) A bar chart of Least Squares Means (LS‐means) of the normalized VAS weight scores. The LS‐means are plotted as a function of BMI centile. The data are grouped separately for parents (PAR) and HCPs (HCP). Error bars represent ±1 standard error of the LS‐mean. (b) A plot of normalized VAS weight scores as a function of participants' comfort scores. Data for successive BMI centiles are colour coded from 2 (brown), 25 (red), 50 (yellow), 75 (green), 91 (blue), 98 (mauve), to 99.6 (dark purple). The slope of the regression of normalized VAS weight score on Comfort score changes systematically from negative at the lowest BMI centiles (brown) to positive at higher BMI centiles (dark purple).

Planned post‐hoc comparisons showed that all successive increments in BMI centile collapsed across participant group and child stimulus type, that is, 2 versus 25, 25 versus 50 and so on, showed statistically significant differences in normalized VAS weight scores at *p* < .05 or less. Further planned post‐hoc comparisons between successive BMI centiles, calculated separately for each participant group (collapsing across child stimulus type), were also all statistically significant at *p* < .01 except for the comparison between BMI centiles 98 versus 99.6, for HCPs. The predicted mean normalized VAS weight scores (Least Squares Means; LS‐means) are shown in Figure 2a. LS‐Means are predicted from the model shown in Supporting Information Table S2. LS‐means are, in effect, within‐group means appropriately adjusted for the other effects in the model.

With respect to covariates in the final model, we found a significant effect of Control (*F*_(1,373)_ = 5.62, *p* = .02) such that the more controllable participants believed child obesity to be by children or their family, so they assigned lower normalized VAS scores. In addition, we found a significant Comfort × BMI centile interaction (*F*_(6,6743)_ = 4.61, *p* = .0001). Participants who reported greater comfort categorizing children as (very) overweight gave the highest VAS weight scores to the heaviest figures and the lowest VAS weight scores to the lowest weight figures. In comparison, participants who were least comfortable assigning higher weight descriptors showed a compressed range of VAS weight scores, and this is illustrated in Figure 2b. Other fixed effects and covariates including participant group, BAS, RSE, TFEQ, overeating, genetic and activity attribution scores, as well as observer gender, age and BMI did not show statistically significant effects and were therefore excluded. The final model explained 67% of the variance in normalized VAS weight scores relative to the unexplained variance in normalized VAS weight scores (Lorah, 2018; Bosker & Snijders, 2011).

While all parents had children under the age of 18, by definition, it proved hard to find HCPs who did not have children under the age of 18. Indeed, 89 HCPs self‐reported having children. This meant that, to some extent, participant group status (i.e., parent versus HCP) was confounded with parenthood per se. In all our analyses (i.e., normalized VAS weight scores, non‐normalized VAS weight scores and weight categorization data), if we included a factor (referred to as CHU18) for whether or not a given participant had a child/children under 18 or not, this factor never made a statistically significant contribution to the models. To explore this issue further, we carried out a sensitivity analysis, which is reported in the Supporting Information.

### Categorization: How accurately could participants categorize images, and which variables were associated with accuracy?

Table 2 displays confusion matrices showing patterns of (mis)match between the centile‐based weight category of the child stimulus (S) and the judgement reported by the participant (R). They illustrate whether judgement congruence was higher for underweight (S1), healthy weight (S2), overweight (S3) or very overweight (S4) images. Cells show the percentage of stimuli in each weight category distributed amongst the 4 response categories, separately for HCPs and parents. Shaded cells indicate congruent responses, that is, the percentage of trials (calculated separately for each row) in which the participant response category matched the centile‐based category (NB: there was a 25% chance of guessing correctly).

**TABLE 2:** Confusion matrices showing the proportion (%) of participant weight judgements that matched the centile‐based category for stimuli (HCPs and parents combined).

	R1	R2	R3	R4
HCPs				
S1	72.3	27.3	.4	.0
S2	9.7	82.7	7.3	.3
S3	.0	51.5	46.3	2.2
S4	.2	23.9	67.2	8.7
Parents				
S1	67.7	32.1	.2	.0
S2	8.8	85.9	5.2	.1
S3	.4	64.3	33.9	1.4
S4	.0	33.6	63.2	3.2

*Note*: 1, underweight; 2, healthy weight; 3, overweight; 4, very overweight.

Abbreviations: R, response; S, stimulus.

Both groups were most likely to correctly assign categories to healthy weight images and much less likely to do so for overweight and very overweight images. Moreover, tests of location for the probability of correct categorization of very overweight stimuli, run separately for HCPs and parents, showed that both groups' performances were significantly lower than guessing (*p* < .0001 in each case).

For the GLMM of a participant's judgement matching the centile‐based category of the figure, Type III tests of fixed effects showed a significant effect for weight category (*F*_(3,6553)_ = 29.04, *p* < .0001), whereby the probability of a correct classification reduced systematically from level 2 to 4 for boys and girls. For images of boys, the probability of a correct response was lower for level 1 than 2, but slightly higher for level 1 than 2 for images of girls. A significant fixed effect of stimulus category (*F*_(3,6553)_ = 17.68, *p* < .0001) was due to a lower probability of a correct categorization for boys (younger *p* = .30, older *p* = .37) and a higher probability for girls (younger *p* = .44, older *p* = .50). In addition, a significant main effect of the participant group (*F*_(1,391)_ = 23.09, *p* < .0001) was due to a higher probability of correct weight classification by HCPs (*p* = .46) compared to parents (*p* = .36).

We found a significant two‐way interactions between weight category × stimulus category (*F*_(9,6553)_ = 29.24, *p* < .0001) owing to the lower probability of correct responses to weight category 1 in boys (younger *p* = .69, older *p* = .56) than girls (younger *p* = .76, older *p* = .83), as well as lower probability responses to weight category 3 in boys (younger *p* = .18, older *p* = .32) than girls (younger *p* = .41, older *p* = .69). A significant interaction between weight category × participant group (*F*_(3,6553)_ = 13.84, *p* < .0001) is attributable to the lower probability of correct responses to weight category 3 by parents (*p* = .32) than HCPs (*p* = .45).

**FIGURE 3:**
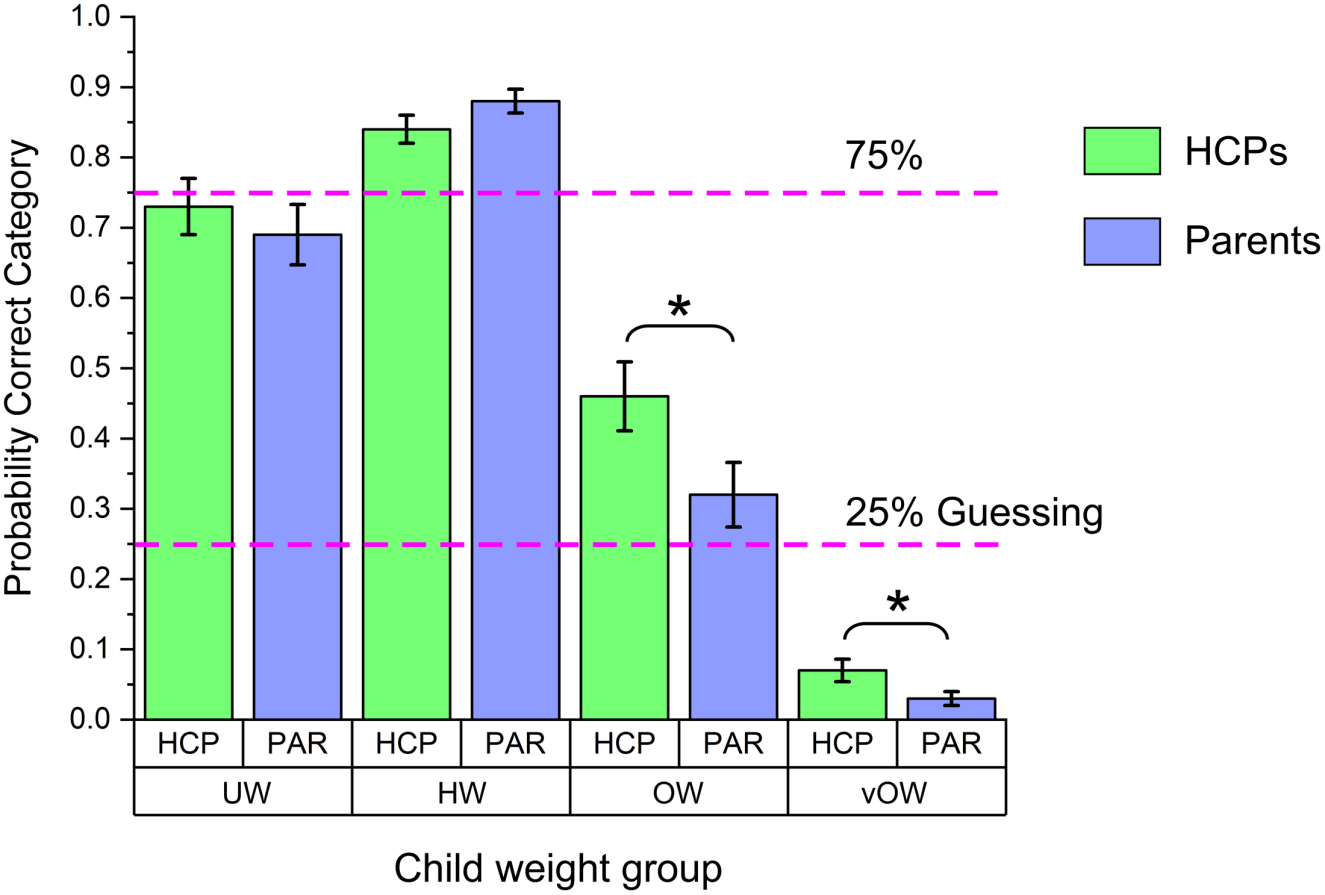
Plots of the model predicted probability of HCPs and parents assigning body to the correct weight category. HW, healthy weight; OW, overweight; UW, underweight; vOW, very overweight.

Planned post‐hoc comparisons showed that all successive increments/decrements in the proportion of correctly assigned weight category, collapsed across the participant group, that is, weight category 1 versus 2, 2 versus 3 and 3 versus 4, were statistically significant at *p* < .005. Additionally, we carried out planned pairwise comparisons between HCPs and Parents as a function of stimulus weight category. As illustrated in Figure 3 we found that HCPs made significantly more correct weight assignments than did the Parents in categories 3 and 4 (i.e., overweight and very overweight), meanwhile parents were significantly better at assigning category 2 (i.e., healthy weight).

**FIGURE 4:**
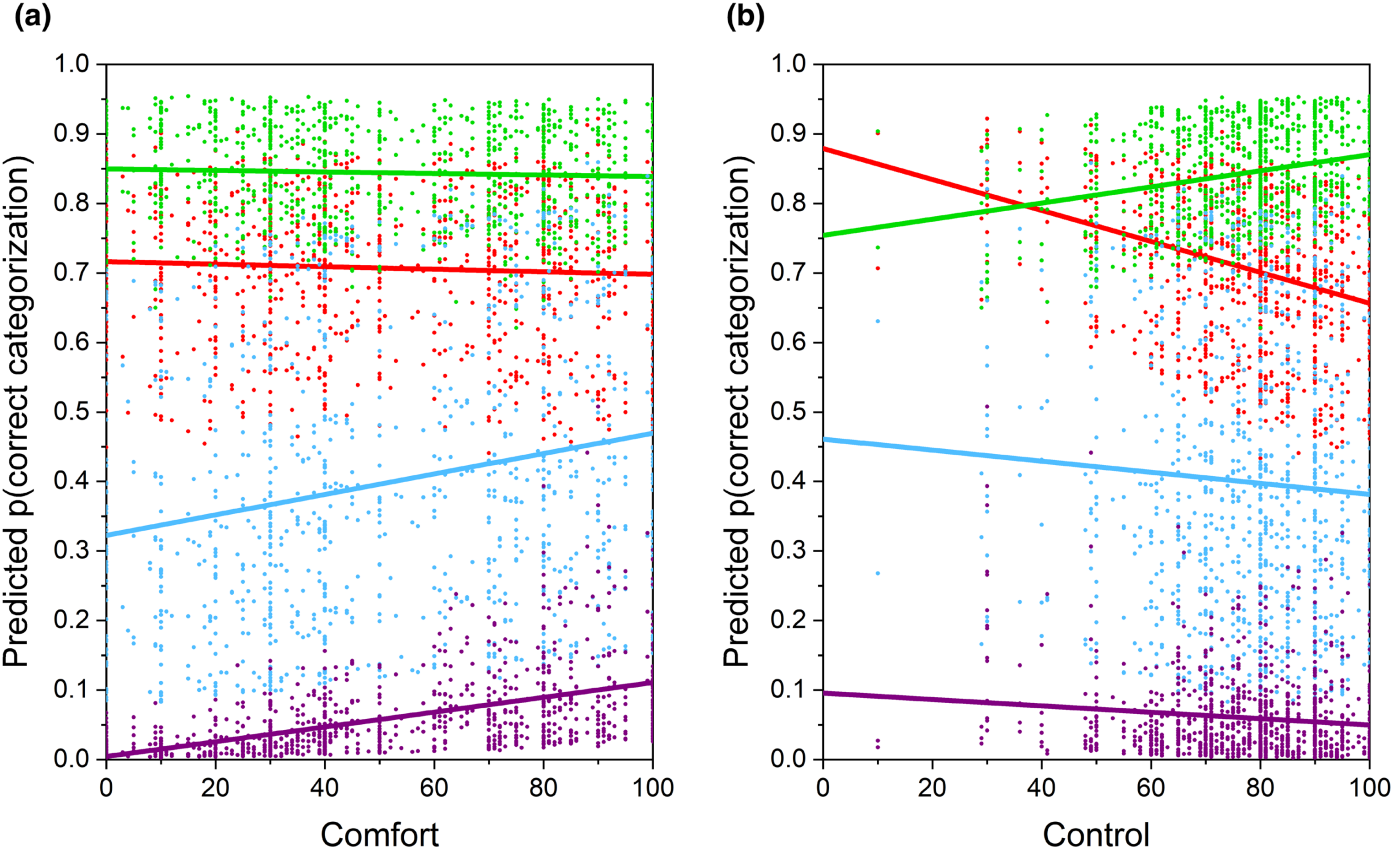
Scatterplots of the probability of a correct categorization predicted from the model in Table S6, plotted as a function of: (a) participants' Comfort scores and (b) participants' Controllability scores. The data for successive stimulus weight categories are colour coded: Underweight (red), healthy weight (green), overweight (blue) and very overweight (dark purple).

With respect to covariates, we found that increasing Comfort (*F*_(1,6553)_ = 27.44, *p* < .0001) scores predicted significant increases in successfully applying higher category labels. Meanwhile increasing Control (*F*_(1,6553)_ = 4.11, *p* = .04) scores predicted significant reductions in successfully applying higher category labels. We also found that the two‐way interactions, weight category × Comfort (*F*_(3,6553)_ = 15.37, *p* < .0001) and weight category × Control (*F*_(3,6553)_ = 6.14, *p* = .0004) were statistically significant. To illustrate these complex effects, Figure 4 shows the predicted probability of correct categorization plotted as a function of participants' Comfort scores (Figure 4a) and Controllability scores (Figure 4b). The slopes of the regressions of the probability of correct categorization on Comfort score change systematically from slightly negative for underweight and healthy weight stimuli to positive for (very) overweight stimuli. Further analysis confirmed that Comfort had no significant effect on underweight and healthy weight stimuli but did have a significant effect on (very) overweight stimuli. This suggests that as participants' Comfort scores increased, they were more likely to correctly assign the overweight and very overweight descriptors. With respect to Controllability, we found that the slopes were most negative for underweight, and less negative for overweight/very overweight stimuli, but positive for healthy weight stimuli. This suggests that stronger belief in the idea that children and parents can control children's weight was associated with higher likelihood of correct assignment of healthy weight and lower likelihood of correct assignment to underweight and overweight/very overweight.

To show the details of the directions of all these effects on categorization, and to see the full model parameters, refer to Tables S5 and S6 of the Supporting Information. Other fixed effects and covariates including participant scores on the BAS, RSE, TFEQ, overeating, genetic, and activity attribution measures, observer sex, age and BMI showed no statistically significant effects and were excluded.

## DISCUSSION

Both parents and HCPs fail to reliably identify higher weight in children, but previous research has not demonstrated *why* (Lundahl et al., 2014). An increased understanding of the determinants of child weight miscategorization could improve detection and treatment of higher child weight by suggesting targets for future intervention (Parkinson et al., 2015). We therefore examined two potential determinants of weight misperception in parents and HCPs: (i) inability to differentiate between child stimuli of different sizes (a perceptual explanation); and (ii) inability or reluctance to assign accurate categorical size descriptors (a cognitive/attitudinal explanation). Our findings ruled out the first explanation and supported the second. These findings are novel: no previous research has concurrently examined these explanations for weight misperception.

### Perceptual factors in child weight judgements

All our participants tended to assign the higher weight bodies to lower weight categories than their BMI centiles would suggest and were least accurate for very overweight stimuli (significantly below chance). This accords with existing research with parents and HCPs in which higher and lower BMI child bodies are miscategorized as being in the recommended weight range (Lundahl et al., 2014; Parry et al., 2008; Queally et al., 2018). Contraction bias and Weber's Law may partially explain this: participants misestimate larger and smaller bodies because these are perceived as being closer in size to bodies in the middle of the scale than they really are, and bodies are increasingly hard to discriminate between as their BMI increases. This narrows the appearance of BMI variation within a scale. This effect likely functions within the broader theoretical context of visual normalization theory, in which the ‘reference’ mid‐point of body size has shifted upwards (Robinson, 2017). Perceptual biases have been documented in a previous study of child weight classification (Evans et al., 2023) and are amenable to alteration (e.g., Harrison & Backus, 2014), though this is effortful. However, visual biases can only be a partial explanation. Participants discriminated between the size increments from one BMI centile to the next using visual analogue scale scores. If visual biases are evident in these data, the effects do not offset participants' ability to make these discriminations between figures (i.e., the participants' perceptual performance shows that the categorization task would be achievable if it only required identification of larger vs. smaller figures). This means that categorization errors are likely to occur for reasons related to the application of specific categorical labels (e.g., very overweight), that is, attitudinal/cognitive.

An additional factor may be the effects of visual diet (Leopold et al., 2001; Winkler & Rhodes, 2005). As we have discussed above, a body's weight status is judged relative to an internal reference or template, which is based on the size of the bodies a person encounters (the ‘visual diet’). This reference body may be used as a yardstick for judging what constitutes a ‘normal’ or ‘average’ body size (Oldham & Robinson, 2017). This reference can be altered in size by seeing more heavy bodies (e.g. Boothroyd et al., 2012; Oldham & Robinson, 2017). Around 26% of children aged 4–5 years and 36% of children aged 10–11 are in the overweight/very overweight range, and this increases to 63.8% in adults (NHS Digital, 2023; Office for Health Improvement & Disparities, 2023). If the reference body has been shifted to a higher BMI by this high proportion of heavier bodies in the visual diet, then this may effectively shift the perceptual weight boundaries to higher levels as the ‘target’ bodies are judged by comparison with this reference. This would mean that participants would effectively underestimate the weight status of heavier bodies, and potentially overestimate the weight status of lower weight bodies.

### Attitudinal beliefs and cognitive factors in child weight judgements

Two attitudinal variables emerged as significant predictors of weight judgements in both tasks: the degree to which participants report (dis)comfort assigning higher weight descriptors to children, and their belief in the controllability of child weight. These effects were seen for both the VAS weight judgements (made using a scale annotated with the NCMP terms) and the categorization task.

This demonstrates for the first time that weight‐related attitudes influence child body judgements, even for computer‐generated figures. First, our findings indicate that weight labels influence body judgements. Participants who reported greater comfort assigning the higher weight descriptors gave the heavier and lighter bodies more extreme VAS weight scores and more accurately assigned the heavier bodies to the (very) overweight categories. Concerns about higher weight descriptors are common and understandable because children and adolescents with higher weights experience weight stigmatization (Pont et al., 2017). Participants may have recognized higher weight amongst the larger figures but may simultaneously seek (consciously or unconsciously) to avoid assigning potentially pejorative terms to children. For example, research has found that there is a counterintuitive association between parents identifying their child as overweight and their subsequent weight (Robinson & Sutin, 2017). It is thought that the stigma associated with being labelled as overweight may partly explain why children whose parents perceive them as overweight are at a greater risk of future weight gain (Robinson & Sutin, 2017). Future studies might investigate whether the use of alternative weight‐related terminology or a health‐focused, rather than weight‐focused, approach would help. This might lead to greater comfort in the recognition of higher weight. Second, a similar effect may account for the effect of controllability scores. Individuals with stronger beliefs in the idea that child weight is controllable may lead them to feel that they would be indirectly blaming a child/parent for their appearance were they to assign the underweight, overweight and very overweight categories. Hence they might feel a reluctance to use such terms, leading to lower accuracy. An additional cognitive explanation, misperception of category boundaries (e.g., the point at which a child's weight enters the ‘(very) overweight’ category), likely applies (cf Poulton, 1989) but was not directly tested in this study. Body judgements seem to be made in a categorical manner, such as ‘healthy’ versus ‘unhealthy’ bodies (Tovée et al., 2012). The position of the boundaries of weight status categories in the BMI centile range was not tested in this study. So, it is not possible to determine if our participants' assignment of weight status is consistent with these boundary positions.

A viable additional/alternative explanation for the observed variation in categorization accuracy may be a general tendency in some individuals to assign all stimuli to a higher weight category (i.e., a generalized shift upwards). This would give an appearance of higher categorization correctness at higher weights, but one would then expect lower correctness for lower weights. We explored this possibility and provided a detailed analysis in Supporting Information Section 3. Overall, our analyses did not support this premise: higher probability of correct categorization for higher weight stimuli was in fact associated with a higher probability of correct categorization for lower weight stimuli.

### Study strengths and weaknesses

This study benefited from the recruitment of adults with regular contact with children and/or potentially pivotal roles in weight identification. Selecting parents of 4‐ to 5‐ and 10‐ to 11‐year‐olds ensured that they were familiar with the age‐groups of children depicted in the stimuli. Recruitment of a wide range of HCPs reflected the multiple roles in which HCPs may encounter children with higher weight. In both cases, parents' and HCPs' weight‐related decision‐making strongly determines whether children receive weight management support or not. Identification of children in need of such support is an essential first step in improving psychological and physical health outcomes, and perception of healthy weight in children with overweight or obesity is a primary barrier to this (Falconer et al., 2014). The scan‐based photorealistic stimuli accurately reflected the variation in adiposity observed across both age groups. Requiring participants to make judgements on these stimuli, rather than reporting the perceived weight status of their own children, ensured standardization of procedure. Our approach also provided the opportunity for participants to be tested on multiple weight‐related decisions in a way that merely asking parents about their own child's weight could not. A key limitation is that parents were not making weight‐related decisions about their own children—as such, this scenario lacked ecological validity. In addition, the study design did not permit inferences about the causal directionality of the relationship between attitudinal measures and categorization patterns.

### Study implications

This study suggests several novel intervention targets to improve recognition of higher weight in children and indicates the importance of actively addressing parental and HCP concerns about the impact of labelling higher weight in children and perceptions of controllability. These concerns have some foundation: critical weight‐loss focused conversations with parents are associated with poorer child self‐esteem and eating disorder symptoms (Gillison et al., 2016). Moreover, in a longitudinal observational study, children *gained* more weight over time when their parents recognized their higher weight category (Robinson & Sutin, 2016). It is therefore imperative that families receive professional weight management support after higher child weight is recognized, rather than recognition itself being the aim. From the HCP perspective, too, support to visually recognize children in need of weight management referral and/or treatment for both higher and lower weights is imperative. HCPs should be made aware that their visual judgements are likely to be inaccurate – simply using objective measures of weight and height on a more regular basis may be an appropriate initial response. In both groups, concerns about the negative impacts of weight categorization should be explicitly discussed and explored when engaging in research co‐design to find practical solutions (Currie et al., 2022), with an acknowledgement of the logic in wanting to protect higher weight children from uncomfortable or negative labelling and from the implication that they or their family should be blamed.

### Conclusions

This study demonstrates a dissociation between the ability to judge the relative size of the bodies within a scale and participants' ability to assign them to the appropriate weight categories. Parents and HCPs were generally able to estimate the relative size of the bodies but were poor at assigning bodies to the lower and upper BMI categories. This pattern of VAS weight results suggests that these categorization errors could not be explained on a purely perceptual basis. Instead, participants' comfort in assigning higher weight categories and their controllability beliefs were also associated with patterns of categorization. Further research should explore these concerns with parents and HCPs in greater depth to ascertain acceptable and feasible ways to support them in making weight‐related decisions about children.

## AUTHOR CONTRIBUTIONS

**Elizabeth H. Evans:** Conceptualization; investigation; writing – review and editing; writing – original draft; funding acquisition. **Bethany J. Ridley:** Methodology; investigation; writing – original draft; writing – review and editing. **Piers L. Cornelissen:** Data curation; formal analysis; visualization; writing – original draft; writing – review and editing. **Robin S. S. Kramer:** Methodology; writing – review and editing. **Vera Araújo‐Soares:** Writing – review and editing. **Martin J. Tovée:** Conceptualization; methodology; writing – original draft; writing – review and editing; funding acquisition.

## FUNDING INFORMATION

This work was supported by the National Institute for Health and Care Research (NIHR127745) and a Northumbria University PhD Studentship.

## CONFLICT OF INTEREST STATEMENT

The authors declare that there is no conflict of interests.

## Supporting information


Data S1.

## Data Availability

The study was pre‐registered at https://doi.org/10.17605/OSF.IO/EWNQ7 and the data are available at https://osf.io/hzunv/.
