# Supplementary material for: Determinants of child body weight categorization in parents and health care professionals: An experimental study
Source: Br J Health Psychol. 2024 Nov 14;30(1):e12765. doi: 10.1111/bjhp.12765 (PMC11586810; doi:10.1111/bjhp.12765)
Supplement: Supplementary file 1 — Data S1. [file BJHP-30-0-s001.docx]

SUPPLEMENTARY MATERIALS

**1. Expansion of the Results**

**1.1 Linear mixed effects model of normalized VAS scores**

Table S1 shows the least-square means (LS-means) computed from the full model whose parameters are shown in Table S2. LS-means are, in effect, within-group means appropriately adjusted for the other effects in the model. The fixed effect of BMI centile is very clear, the LS-mean normalized VAS scores increase systematically as a function of BMI centile. The fixed effect of stimulus type is attributable to the higher LS-means for girls (older M = 57.3, younger = 57.3) than for boys (older M = 55.9, younger = 51.5). The significant participant group × BMI centile interaction is due to the narrower range of LS-mean normalized-VAS scores for HCPs (16.0 – 80.4) than it is for parents (13.7 – 84.8) across BMI centiles. The significant stimulus type × BMI centile interaction is due to the narrower range of LS-means for boys (older = 24.7 – 64.5, younger = 16.2 – 76.7) than for girls (older = 8.0 – 83.4, younger = 10.5 – 85.4) across BMI centiles. The significant three-way interaction participant group × BMI centile × stimulus type is due to the fact that the wider range of LS-means for girls, across the BMI centiles, is larger for parents than for HCPs.

Table S1 Table of LS-Means for normalized VAS score from the LME model

| HCP |  |  |  |  |  |  | PARENTS |  |  |  |
| --- | --- | --- | --- | --- | --- | --- | --- | --- | --- | --- |
| image | BMIc | VASnorm | 95% CI | | |  | VASnorm | 95% CI | | |
| BO | 2 | 24.45 | 21.61 | - | 27.28 |  | 24.86 | 21.82 | - | 27.89 |
| BO | 25 | 39.69 | 36.87 | - | 42.52 |  | 42.78 | 39.75 | - | 45.82 |
| BO | 50 | 48.58 | 45.76 | - | 51.41 |  | 45.89 | 42.86 | - | 48.93 |
| BO | 75 | 52.82 | 49.99 | - | 55.64 |  | 50.87 | 47.83 | - | 53.90 |
| BO | 91 | 63.60 | 60.77 | - | 66.42 |  | 65.50 | 62.46 | - | 68.53 |
| BO | 98 | 79.91 | 77.08 | - | 82.74 |  | 78.83 | 75.80 | - | 81.87 |
| BO | 99.6 | 82.16 | 79.33 | - | 84.99 |  | 83.06 | 80.02 | - | 86.09 |
|  | mean | 55.89 |  |  |  |  | 55.97 |  |  |  |
|  |  |  |  |  |  |  |  |  |  |  |
| BY | 2 | 15.68 | 12.80 | - | 18.55 |  | 16.82 | 13.95 | - | 19.68 |
| BY | 25 | 36.40 | 33.52 | - | 39.29 |  | 37.36 | 34.49 | - | 40.22 |
| BY | 50 | 45.60 | 42.72 | - | 48.47 |  | 45.72 | 42.85 | - | 48.59 |
| BY | 75 | 50.39 | 47.51 | - | 53.26 |  | 51.05 | 48.18 | - | 53.91 |
| BY | 91 | 58.01 | 55.14 | - | 60.89 |  | 61.96 | 59.09 | - | 64.82 |
| BY | 98 | 73.27 | 70.40 | - | 76.14 |  | 76.03 | 73.16 | - | 78.89 |
| BY | 99.6 | 73.94 | 71.07 | - | 76.82 |  | 79.36 | 76.50 | - | 82.23 |
|  | mean | 50.47 |  |  |  |  | 52.61 |  |  |  |
|  |  |  |  |  |  |  |  |  |  |  |
| GO | 2 | 11.46 | 8.80 | - | 14.12 |  | 4.59 | 1.55 | - | 7.62 |
| GO | 25 | 34.36 | 31.71 | - | 37.01 |  | 29.94 | 26.90 | - | 32.97 |
| GO | 50 | 51.29 | 48.63 | - | 53.94 |  | 51.51 | 48.48 | - | 54.54 |
| GO | 75 | 59.62 | 56.96 | - | 62.27 |  | 59.64 | 56.60 | - | 62.67 |
| GO | 91 | 81.16 | 78.51 | - | 83.81 |  | 80.64 | 77.60 | - | 83.67 |
| GO | 98 | 82.20 | 79.55 | - | 84.86 |  | 84.65 | 81.62 | - | 87.69 |
| GO | 99.6 | 83.53 | 80.88 | - | 86.19 |  | 87.61 | 84.58 | - | 90.64 |
|  | mean | 57.66 |  |  |  |  | 56.94 |  |  |  |
|  |  |  |  |  |  |  |  |  |  |  |
| GY | 2 | 12.59 | 9.86 | - | 15.32 |  | 8.45 | 5.58 | - | 11.31 |
| GY | 25 | 32.40 | 29.68 | - | 35.13 |  | 34.49 | 31.62 | - | 37.35 |
| GY | 50 | 53.11 | 50.38 | - | 55.84 |  | 54.74 | 51.87 | - | 57.61 |
| GY | 75 | 59.26 | 56.54 | - | 61.99 |  | 62.76 | 59.90 | - | 65.63 |
| GY | 91 | 67.36 | 64.64 | - | 70.09 |  | 70.39 | 67.52 | - | 73.25 |
| GY | 98 | 81.82 | 79.08 | - | 84.55 |  | 88.98 | 86.12 | - | 91.85 |
| GY | 99.6 | 84.69 | 81.95 | - | 87.42 |  | 90.72 | 87.86 | - | 93.59 |
|  | mean | 55.89 |  |  |  |  | 58.65 |  |  |  |

NB BY = younger boys, BO = older boys, GY = younger girls, and GO = older girls.

The following is intended to help interpretation of Table S2, which contains the linear mixed effects model parameters. The explanatory variables: participant group (i.e., grp: HCP, PAR), stimulus type (i.e., image: BO, BY, GO, GY), and BMI centile (i.e., bmic: 2, 25, 50, 75, 91, 98, and 99.6) were coded as categories. Therefore, they were dummy coded with PAR, GY, and 99.6 acting as controls for grp, image and bmic, respectively. In Table S2, the simplest fixed effects, such as grp, bmic, and image contribute 2, 7, and 4 rows, respectively. In each case, the parameter estimates given represent the difference between the control for a given categorical variable, for example GY for image, and another factor level, GO, for example (i.e., -1.37 in Table S2). The t-test indicates whether this difference is statistically significant at p<.05. The variables Comfort and Control are continuous variables whose parameter estimates are regression weights. Their t-tests indicate whether these regression weights are significantly different from zero.

Table S2 LME parameters from normalized VAS data

| Effect | grp | image | bmic | Estimate | SE | t Value (DF) | p value | Lower |  | Upper |
| --- | --- | --- | --- | --- | --- | --- | --- | --- | --- | --- |
| Intercept |  |  |  | 90.65 | 2.88 | 31.45 (651) | <.0001 | 84.99 | - | 96.31 |
| grp | HCP |  |  | -7.17 | 2.02 | -3.54 (3640) | 0.0004 | -11.13 | - | -3.20 |
| grp | PAR |  |  | 0.00 | . | . | . | . |  | . |
| bmic |  |  | 2 | -75.57 | 2.11 | -35.89 (6743) | <.0001 | -79.70 | - | -71.44 |
| bmic |  |  | 25 | -50.58 | 2.11 | -24.02 (6743) | <.0001 | -54.70 | - | -46.45 |
| bmic |  |  | 50 | -30.66 | 2.11 | -14.56 (6743) | <.0001 | -34.78 | - | -26.53 |
| bmic |  |  | 75 | -23.51 | 2.11 | -11.17 (6743) | <.0001 | -27.64 | - | -19.38 |
| bmic |  |  | 91 | -17.14 | 2.11 | -8.14 (6743) | <.0001 | -21.27 | - | -13.014 |
| bmic |  |  | 98 | 3.76 | 2.11 | 1.79 (6743) | 0.07 | -0.37 | - | 7.89 |
| bmic |  |  | 99.6 | 0.00 | . | . | . | . |  | . |
| image |  | BO |  | -5.93 | 2.13 | -2.79 (3646) | 0.01 | -10.10 | - | -1.76 |
| image |  | BY |  | -9.62 | 1.81 | -5.31 (6743) | <.0001 | -13.18 | - | -6.07 |
| image |  | GO |  | -1.37 | 2.13 | -0.65 (3646) | 0.5 | -5.54 | - | 2.79 |
| image |  | GY |  | 0.00 | . | . | . | . |  | . |
| grp × bmic | HCP |  | 2 | 11.31 | 2.50 | 4.52 (6744) | <.0001 | 6.40 | - | 16.22 |
| grp × bmic | HCP |  | 25 | 5.082 | 2.50 | 2.030 (6743) | 0.04 | 0.18 | - | 9.99 |
| grp × bmic | HCP |  | 50 | 5.54 | 2.50 | 2.21 (6744) | 0.03 | 0.63 | - | 10.45 |
| grp × bmic | HCP |  | 75 | 3.67 | 2.50 | 1.47 (6743) | 0.14 | -1.24 | - | 8.57 |
| grp × bmic | HCP |  | 91 | 4.14 | 2.50 | 1.66 (6743) | 0.10 | -0.76 | - | 9.045 |
| grp × bmic | HCP |  | 98 | 1.13 | 2.50 | 0.45 (6743) | 0.65 | -3.78 | - | 6.040 |
| grp × bmic | HCP |  | 99.6 | 0 | . | . | . | . |  | . |
| grp × bmic | PAR |  | 2 | 0 | . | . | . | . |  | . |
| grp × bmic | PAR |  | 25 | 0 | . | . | . | . |  | . |
| grp × bmic | PAR |  | 50 | 0 | . | . | . | . |  | . |
| grp × bmic | PAR |  | 75 | 0 | . | . | . | . |  | . |
| grp × bmic | PAR |  | 91 | 0 | . | . | . | . |  | . |
| grp × bmic | PAR |  | 98 | 0 | . | . | . | . |  | . |
| grp × bmic | PAR |  | 99.6 | 0 | . | . | . | . |  | . |
| image × bmic |  | BO | 2 | 22.34 | 2.64 | 8.46 (6743) | <.0001 | 17.16 | - | 27.52 |
| image × bmic |  | BO | 25 | 14.22 | 2.64 | 5.38 (6743) | <.0001 | 9.046 | - | 19.40 |
| image × bmic |  | BO | 50 | -2.92 | 2.64 | -1.11 (6743) | 0.3 | -8.10 | - | 2.26 |
| image × bmic |  | BO | 75 | -5.97 | 2.64 | -2.26 (6743) | 0.0 | -11.15 | - | -0.79 |
| image × bmic |  | BO | 91 | 1.04 | 2.64 | 0.39 (6743) | 0.7 | -4.14 | - | 6.21 |
| image × bmic |  | BO | 98 | -5.96 | 2.64 | -2.26 (6743) | 0.0 | -11.14 | - | -0.78 |
| image × bmic |  | BO | 99.6 | 0.00 | . | . | . | . |  | . |
| image × bmic |  | BY | 2 | 17.99 | 2.56 | 7.02 (6743) | <.0001 | 12.97 | - | 23.02 |
| image × bmic |  | BY | 25 | 12.49 | 2.56 | 4.87 (6743) | <.0001 | 7.47 | - | 17.52 |
| image × bmic |  | BY | 50 | 0.60 | 2.56 | 0.23 (6743) | 0.8 | -4.42 | - | 5.63 |
| image × bmic |  | BY | 75 | -2.094 | 2.56 | -0.82 (6743) | 0.4 | -7.12 | - | 2.93 |
| image × bmic |  | BY | 91 | 1.19 | 2.56 | 0.47 (6743) | 0.6 | -3.83 | - | 6.22 |
| image × bmic |  | BY | 98 | -5.073 | 2.56 | -1.98 (6743) | 0.05 | -10.10 | - | -0.048 |
| image × bmic |  | BY | 99.6 | 0.00 | . | . | . | . |  | . |
| image × bmic |  | GO | 2 | -2.49 | 2.64 | -0.94 (6743) | 0.3 | -7.67 | - | 2.69 |
| image × bmic |  | GO | 25 | -3.18 | 2.64 | -1.20 (6743) | 0.2 | -8.35 | - | 2.00 |
| image × bmic |  | GO | 50 | -1.86 | 2.64 | -0.70 (6743) | 0.5 | -7.03 | - | 3.32 |
| image × bmic |  | GO | 75 | -1.75 | 2.64 | -0.66 (6743) | 0.5 | -6.93 | - | 3.43 |
| image × bmic |  | GO | 91 | 11.62 | 2.64 | 4.40 (6743) | <.0001 | 6.45 | - | 16.80 |
| image × bmic |  | GO | 98 | -4.69 | 2.64 | -1.78 (6743) | 0.08 | -9.87 | - | 0.48 |
| image × bmic |  | GO | 99.6 | 0 | . | . | . | . |  | . |
| image × bmic |  | GY | 2 | 0 | . | . | . | . |  | . |
| image × bmic |  | GY | 25 | 0 | . | . | . | . |  | . |
| image × bmic |  | GY | 50 | 0 | . | . | . | . |  | . |
| image × bmic |  | GY | 75 | 0 | . | . | . | . |  | . |
| image × bmic |  | GY | 91 | 0 | . | . | . | . |  | . |
| image × bmic |  | GY | 98 | 0 | . | . | . | . |  | . |
| image × bmic |  | GY | 99.6 | 0 | . | . | . | . |  | . |
| grp × image × bmic | HCP | BO | 2 | -4.55 | 2.78 | -1.64 (5798) | 0.1 | -10.00 | - | 0.89 |
| grp × image × bmic | HCP | BO | 25 | -1.01 | 2.77 | -0.36 (5788) | 0.7 | -6.44 | - | 4.43 |
| grp × image × bmic | HCP | BO | 50 | 4.32 | 2.78 | 1.56 (5793) | 0.1 | -1.12 | - | 9.77 |
| grp × image × bmic | HCP | BO | 75 | 5.45 | 2.77 | 1.97 (5788) | 0.05 | 0.013 | - | 10.89 |
| grp × image × bmic | HCP | BO | 91 | 1.13 | 2.77 | 0.41 (5788) | 0.7 | -4.31 | - | 6.57 |
| grp × image × bmic | HCP | BO | 98 | 7.11 | 2.78 | 2.56 (5793) | 0.01 | 1.67 | - | 12.55 |
| grp × image × bmic | HCP | BO | 99.6 | 6.27 | 2.78 | 2.26 (5793) | 0.02 | 0.83 | - | 11.72 |
| grp × image × bmic | HCP | BY | 2 | -5.28 | 2.55 | -2.07 (6746) | 0.04 | -10.28 | - | -0.29 |
| grp × image × bmic | HCP | BY | 25 | 1.13 | 2.55 | 0.44 (6746) | 0.7 | -3.86 | - | 6.13 |
| grp × image × bmic | HCP | BY | 50 | 1.51 | 2.55 | 0.59 (6746) | 0.6 | -3.49 | - | 6.50 |
| grp × image × bmic | HCP | BY | 75 | 2.84 | 2.55 | 1.12 (6746) | 0.3 | -2.15 | - | 7.83 |
| grp × image × bmic | HCP | BY | 91 | -0.92 | 2.55 | -0.36 (6746) | 0.7 | -5.91 | - | 4.071 |
| grp × image × bmic | HCP | BY | 98 | 3.28 | 2.55 | 1.29 (6746) | 0.2 | -1.72 | - | 8.27 |
| grp × image × bmic | HCP | BY | 99.6 | 1.75 | 2.55 | 0.69 (6746) | 0.5 | -3.25 | - | 6.74 |
| grp × image × bmic | HCP | GO | 2 | 2.73 | 2.74 | 1.00 (5678) | 0.3 | -2.63 | - | 8.10 |
| grp × image × bmic | HCP | GO | 25 | 6.51 | 2.73 | 2.38 (5669) | 0.02 | 1.15 | - | 11.87 |
| grp × image × bmic | HCP | GO | 50 | 1.41 | 2.73 | 0.51 (5674) | 0.6 | -3.96 | - | 6.77 |
| grp × image × bmic | HCP | GO | 75 | 3.48 | 2.73 | 1.27 (5669) | 0.2 | -1.88 | - | 8.84 |
| grp × image × bmic | HCP | GO | 91 | 3.55 | 2.73 | 1.30 (5669) | 0.2 | -1.81 | - | 8.91 |
| grp × image × bmic | HCP | GO | 98 | 3.58 | 2.73 | 1.31 (5674) | 0.2 | -1.78 | - | 8.94 |
| grp × image × bmic | HCP | GO | 99.6 | 3.09 | 2.73 | 1.13 (5674) | 0.3 | -2.27 | - | 8.45 |
| grp × image × bmic | HCP | GY | 2 | 0 | . | . | . | . |  | . |
| grp × image × bmic | HCP | GY | 25 | 0 | . | . | . | . |  | . |
| grp × image × bmic | HCP | GY | 50 | 0 | . | . | . | . |  | . |
| grp × image × bmic | HCP | GY | 75 | 0 | . | . | . | . |  | . |
| grp × image × bmic | HCP | GY | 91 | 0 | . | . | . | . |  | . |
| grp × image × bmic | HCP | GY | 98 | 0 | . | . | . | . |  | . |
| grp × image × bmic | HCP | GY | 99.6 | 0 | . | . | . | . |  | . |
| grp × image × bmic | PAR | BO | 2 | 0 | . | . | . | . |  | . |
| grp × image × bmic | PAR | BO | 25 | 0 | . | . | . | . |  | . |
| grp × image × bmic | PAR | BO | 50 | 0 | . | . | . | . |  | . |
| grp × image × bmic | PAR | BO | 75 | 0 | . | . | . | . |  | . |
| grp × image × bmic | PAR | BO | 91 | 0 | . | . | . | . |  | . |
| grp × image × bmic | PAR | BO | 98 | 0 | . | . | . | . |  | . |
| grp × image × bmic | PAR | BO | 99.6 | 0 | . | . | . | . |  | . |
| grp × image × bmic | PAR | BY | 2 | 0 | . | . | . | . |  | . |
| grp × image × bmic | PAR | BY | 25 | 0 | . | . | . | . |  | . |
| grp × image × bmic | PAR | BY | 50 | 0 | . | . | . | . |  | . |
| grp × image × bmic | PAR | BY | 75 | 0 | . | . | . | . |  | . |
| grp × image × bmic | PAR | BY | 91 | 0 | . | . | . | . |  | . |
| grp × image × bmic | PAR | BY | 98 | 0 | . | . | . | . |  | . |
| grp × image × bmic | PAR | BY | 99.6 | 0 | . | . | . | . |  | . |
| grp × image × bmic | PAR | GO | 2 | 0 | . | . | . | . |  | . |
| grp × image × bmic | PAR | GO | 25 | 0 | . | . | . | . |  | . |
| grp × image × bmic | PAR | GO | 50 | 0 | . | . | . | . |  | . |
| grp × image × bmic | PAR | GO | 75 | 0 | . | . | . | . |  | . |
| grp × image × bmic | PAR | GO | 91 | 0 | . | . | . | . |  | . |
| grp × image × bmic | PAR | GO | 98 | 0 | . | . | . | . |  | . |
| grp × image × bmic | PAR | GO | 99.6 | 0 | . | . | . | . |  | . |
| grp × image × bmic | PAR | GY | 2 | 0 | . | . | . | . |  | . |
| grp × image × bmic | PAR | GY | 25 | 0 | . | . | . | . |  | . |
| grp × image × bmic | PAR | GY | 50 | 0 | . | . | . | . |  | . |
| grp × image × bmic | PAR | GY | 75 | 0 | . | . | . | . |  | . |
| grp × image × bmic | PAR | GY | 91 | 0 | . | . | . | . |  | . |
| grp × image × bmic | PAR | GY | 98 | 0 | . | . | . | . |  | . |
| grp × image × bmic | PAR | GY | 99.6 | 0 | . | . | . | . |  | . |
| Comfort |  |  |  | 0.077 | 0.020 | 3.77 (1271) | 0.0002 | 0.037 | - | 0.12 |
| Comfort × bmic |  |  | 2 | -0.096 | 0.021 | -4.52 (6744) | <.0001 | -0.14 | - | -0.054 |
| Comfort × bmic |  |  | 25 | -0.076 | 0.021 | -3.57 (6743) | 0.0004 | -0.12 | - | -0.034 |
| Comfort × bmic |  |  | 50 | -0.069 | 0.021 | -3.27 (6743) | 0.001 | -0.11 | - | -0.028 |
| Comfort × bmic |  |  | 75 | -0.052 | 0.021 | -2.47 (6743) | 0.01 | -0.09 | - | -0.011 |
| Comfort × bmic |  |  | 91 | -0.028 | 0.021 | -1.32 (6743) | 0.2 | -0.070 | - | 0.013 |
| Comfort × bmic |  |  | 98 | -0.039 | 0.021 | -1.84 (6743) | 0.1 | -0.081 | - | 0.0025 |
| Comfort × bmic |  |  | 99.6 | 0.000 | . | . | . | . |  | . |
| Control |  |  |  | -0.073 | 0.031 | -2.37 (373) | 0.02 | -0.13 | - | -0.012 |

**1.2 Linear mixed effects model of non-normalized VAS scores**

Table S3 shows the least-square means (LS-means) computed from an equivalent model of the non-normalized VAS scores, whose parameters are shown in Table S4. The results are very similar to those shown in Table S1. The main differences are that the ranges for non-normalized VAS scores are overall narrower than those of the normalized VAS scores. In addition, the three-way interaction group × BMI centile × stimulus was not statistically significant for the non-normalized VAS data. Finally, Table S4 shows that we found significant effects of rater sex (i.e., obsex) and genetic attribution scores (i.e., genetic) for the non-normalized data. Female raters assigned higher VAS scores (LS-mean = 53.1) than male raters (LS-mean = 51.1), and as raters’ genetic attribution scores increased, so the VAS scores they assigned systematically decreased.

Table S3 Table of LS-Means for non-normalized VAS score from an LME model

|  |  | HCP |  |  |  |  | PARENTS | |  |  |
| --- | --- | --- | --- | --- | --- | --- | --- | --- | --- | --- |
| image | BMIc | VAS | 95% CI | | |  | VAS | 95% CI | | |
| BO | 2 | 36.83 | 35.20 | - | 38.46 |  | 38.86 | 37.18 | - | 40.54 |
| BO | 25 | 45.01 | 43.39 | - | 46.64 |  | 46.71 | 45.03 | - | 48.39 |
| BO | 50 | 49.32 | 47.70 | - | 50.95 |  | 47.93 | 46.25 | - | 49.60 |
| BO | 75 | 51.63 | 50.01 | - | 53.26 |  | 50.12 | 48.44 | - | 51.80 |
| BO | 91 | 56.70 | 55.07 | - | 58.32 |  | 56.21 | 54.53 | - | 57.89 |
| BO | 98 | 64.35 | 62.72 | - | 65.98 |  | 61.73 | 60.05 | - | 63.40 |
| BO | 99.6 | 65.71 | 64.08 | - | 67.33 |  | 63.43 | 61.76 | - | 65.11 |
|  | mean | 52.79 |  |  |  |  | 52.14 |  |  |  |
|  |  |  |  |  |  |  |  |  |  |  |
| BY | 2 | 33.26 | 31.60 | - | 34.91 |  | 35.55 | 33.98 | - | 37.12 |
| BY | 25 | 43.47 | 41.81 | - | 45.13 |  | 43.91 | 42.34 | - | 45.48 |
| BY | 50 | 47.93 | 46.28 | - | 49.58 |  | 47.05 | 45.48 | - | 48.62 |
| BY | 75 | 50.56 | 48.90 | - | 52.21 |  | 49.10 | 47.54 | - | 50.67 |
| BY | 91 | 54.27 | 52.62 | - | 55.92 |  | 53.34 | 51.77 | - | 54.91 |
| BY | 98 | 61.22 | 59.56 | - | 62.87 |  | 59.26 | 57.69 | - | 60.83 |
| BY | 99.6 | 62.01 | 60.36 | - | 63.66 |  | 60.24 | 58.68 | - | 61.81 |
|  | mean | 50.39 |  |  |  |  | 49.78 |  |  |  |
|  |  |  |  |  |  |  |  |  |  |  |
| GO | 2 | 30.72 | 29.19 | - | 32.25 |  | 30.20 | 28.52 | - | 31.88 |
| GO | 25 | 41.95 | 40.43 | - | 43.48 |  | 41.35 | 39.67 | - | 43.03 |
| GO | 50 | 51.48 | 49.96 | - | 53.01 |  | 50.43 | 48.75 | - | 52.11 |
| GO | 75 | 55.18 | 53.65 | - | 56.70 |  | 53.74 | 52.06 | - | 55.42 |
| GO | 91 | 65.29 | 63.77 | - | 66.82 |  | 62.58 | 60.90 | - | 64.26 |
| GO | 98 | 66.05 | 64.52 | - | 67.57 |  | 63.89 | 62.21 | - | 65.57 |
| GO | 99.6 | 66.82 | 65.29 | - | 68.34 |  | 65.30 | 63.62 | - | 66.97 |
|  | mean | 53.93 |  |  |  |  | 52.50 |  |  |  |
|  |  |  |  |  |  |  |  |  |  |  |
| GY | 2 | 31.44 | 29.87 | - | 33.01 |  | 31.63 | 30.06 | - | 33.20 |
| GY | 25 | 41.43 | 39.86 | - | 42.99 |  | 41.68 | 40.11 | - | 43.25 |
| GY | 50 | 51.68 | 50.10 | - | 53.25 |  | 50.77 | 49.20 | - | 52.34 |
| GY | 75 | 54.88 | 53.31 | - | 56.44 |  | 54.01 | 52.44 | - | 55.58 |
| GY | 91 | 58.96 | 57.39 | - | 60.52 |  | 56.57 | 55.00 | - | 58.14 |
| GY | 98 | 66.33 | 64.76 | - | 67.90 |  | 64.43 | 62.86 | - | 65.99 |
| GY | 99.6 | 67.67 | 66.10 | - | 69.24 |  | 64.90 | 63.33 | - | 66.47 |
|  | mean | 53.20 |  |  |  |  | 52.00 |  |  |  |

NB BY = younger boys, BO = older boys, GY = younger girls, and GO = older girls.

Table S4 LME parameters from non-normalized VAS data

| Effect | grp | image | obsex | bmic | Estimate | SE | t value (DF) | p value | Lower |  | Upper |
| --- | --- | --- | --- | --- | --- | --- | --- | --- | --- | --- | --- |
| Intercept |  |  |  |  | 56.25 | 2.21 | 25.42 (984) | <.0001 | 51.91 | - | 60.60 |
| grp | HCP |  |  |  | 1.89 | 0.71 | 2.65 (1172) | 0.0081 | 0.49 | - | 3.28 |
| grp | PAR |  |  |  | 0.00 | . | . | . | . |  | . |
| bmic |  |  |  | 2 | -27.40 | 2.09 | -13.12 (6719) | <.0001 | -31.50 | - | -23.31 |
| bmic |  |  |  | 25 | -18.31 | 2.09 | -8.77 (6719) | <.0001 | -22.40 | - | -14.21 |
| bmic |  |  |  | 50 | -9.77 | 2.09 | -4.68 (6719) | <.0001 | -13.86 | - | -5.68 |
| bmic |  |  |  | 75 | -7.63 | 2.09 | -3.65 (6719) | 0.0003 | -11.72 | - | -3.54 |
| bmic |  |  |  | 91 | -5.89 | 2.09 | -2.82 (6719) | 0.0048 | -9.98 | - | -1.79 |
| bmic |  |  |  | 98 | 1.76 | 2.09 | 0.84 (6719) | 0.3987 | -2.33 | - | 5.86 |
| bmic |  |  |  | 99.6 | 0.00 | . | . | . | . |  | . |
| image |  | BO |  |  | -0.75 | 0.74 | -1.02 (7016) | 0.3101 | -2.21 | - | 0.70 |
| image |  | BY |  |  | -4.25 | 0.71 | -5.99 (6722) | <.0001 | -5.64 | - | -2.86 |
| image |  | GO |  |  | 0.70 | 0.73 | 0.96 (7033) | 0.3392 | -0.73 | - | 2.13 |
| image |  | GY |  |  | 0.00 | . | . | . | . |  | . |
| grp × bmic | HCP |  |  | 2 | -2.80 | 0.73 | -3.85 (6720) | 0.0001 | -4.22 | - | -1.37 |
| grp × bmic | HCP |  |  | 25 | -2.34 | 0.73 | -3.21 (6719) | 0.0013 | -3.76 | - | -0.91 |
| grp × bmic | HCP |  |  | 50 | -0.87 | 0.73 | -1.20 (6719) | 0.2319 | -2.29 | - | 0.56 |
| grp × bmic | HCP |  |  | 75 | -0.59 | 0.73 | -0.81 (6719) | 0.4167 | -2.01 | - | 0.83 |
| grp × bmic | HCP |  |  | 91 | -0.21 | 0.73 | -0.28 (6719) | 0.7767 | -1.63 | - | 1.22 |
| grp × bmic | HCP |  |  | 98 | 0.50 | 0.73 | 0.69 (6719) | 0.4915 | -0.92 | - | 1.92 |
| grp × bmic | HCP |  |  | 99.6 | 0.00 | . | . | . | . |  | . |
| grp × bmic | PAR |  |  | 2 | 0.00 | . | . | . | . |  | . |
| grp × bmic | PAR |  |  | 25 | 0.00 | . | . | . | . |  | . |
| grp × bmic | PAR |  |  | 50 | 0.00 | . | . | . | . |  | . |
| grp × bmic | PAR |  |  | 75 | 0.00 | . | . | . | . |  | . |
| grp × bmic | PAR |  |  | 91 | 0.00 | . | . | . | . |  | . |
| grp × bmic | PAR |  |  | 98 | 0.00 | . | . | . | . |  | . |
| grp × bmic | PAR |  |  | 99.6 | 0.00 | . | . | . | . |  | . |
| image × bmic |  | BO |  | 2 | 7.094 | 1.02 | 6.98 (6719) | <.0001 | 5.10 | - | 9.09 |
| image × bmic |  | BO |  | 25 | 5.073 | 1.02 | 4.99 (6719) | <.0001 | 3.08 | - | 7.06 |
| image × bmic |  | BO |  | 50 | -1.80 | 1.02 | -1.77 (6719) | 0.0763 | -3.80 | - | 0.19 |
| image × bmic |  | BO |  | 75 | -2.77 | 1.02 | -2.72 (6719) | 0.0065 | -4.76 | - | -0.77 |
| image × bmic |  | BO |  | 91 | -0.54 | 1.02 | -0.53 (6719) | 0.5953 | -2.53 | - | 1.45 |
| image × bmic |  | BO |  | 98 | -2.45 | 1.02 | -2.41 (6719) | 0.016 | -4.44 | - | -0.46 |
| image × bmic |  | BO |  | 99.6 | 0.00 | . | . | . | . |  | . |
| image × bmic |  | BY |  | 2 | 7.15 | 1.0030 | 7.13 (6719) | <.0001 | 5.18 | - | 9.12 |
| image × bmic |  | BY |  | 25 | 6.39 | 1.0032 | 6.37 (6719) | <.0001 | 4.42 | - | 8.35 |
| image × bmic |  | BY |  | 50 | 0.52 | 1.0031 | 0.52 (6719) | 0.6031 | -1.44 | - | 2.49 |
| image × bmic |  | BY |  | 75 | -0.37 | 1.0026 | -0.36 (6719) | 0.7158 | -2.33 | - | 1.60 |
| image × bmic |  | BY |  | 91 | 0.30 | 1.0026 | 0.30 (6719) | 0.7611 | -1.66 | - | 2.27 |
| image × bmic |  | BY |  | 98 | -1.79 | 1.0030 | -1.79 (6719) | 0.074 | -3.76 | - | 0.17 |
| image × bmic |  | BY |  | 99.6 | 0.00 | . | . | . | . |  | . |
| image × bmic |  | GO |  | 2 | -1.64 | 0.99 | -1.65 (6719) | 0.099 | -3.59 | - | 0.31 |
| image × bmic |  | GO |  | 25 | -0.51 | 0.99 | -0.51 (6719) | 0.6102 | -2.45 | - | 1.44 |
| image × bmic |  | GO |  | 50 | -0.93 | 0.99 | -0.94 (6719) | 0.3463 | -2.88 | - | 1.01 |
| image × bmic |  | GO |  | 75 | -0.64 | 0.99 | -0.64 (6719) | 0.5202 | -2.58 | - | 1.31 |
| image × bmic |  | GO |  | 91 | 5.57 | 0.99 | 5.62 (6719) | <.0001 | 3.63 | - | 7.52 |
| image × bmic |  | GO |  | 98 | -2.00 | 0.99 | -2.01 (6719) | 0.0443 | -3.94 | - | -0.05 |
| image × bmic |  | GO |  | 99.6 | 0.00 | . | . | . | . |  | . |
| image × bmic |  | GY |  | 2 | 0.00 | . | . | . | . |  | . |
| image × bmic |  | GY |  | 25 | 0.00 | . | . | . | . |  | . |
| image × bmic |  | GY |  | 50 | 0.00 | . | . | . | . |  | . |
| image × bmic |  | GY |  | 75 | 0.00 | . | . | . | . |  | . |
| image × bmic |  | GY |  | 91 | 0.00 | . | . | . | . |  | . |
| image × bmic |  | GY |  | 98 | 0.00 | . | . | . | . |  | . |
| image × bmic |  | GY |  | 99.6 | 0.00 | . | . | . | . |  | . |
| obsex |  |  | F |  | 1.93 | 0.53 | 3.62 (390) | 0.0003 | 0.88 | - | 2.98 |
| obsex |  |  | M |  | 0.00 | . | . | . | . |  | . |
| genetic |  |  |  |  | -0.022 | 0.011 | -1.95 (379) | 0.0514 | -0.044 | - | 0.00013 |
| Comfort |  |  |  |  | 0.063 | 0.012 | 5.40 (1366) | <.0001 | 0.040 | - | 0.086 |
| Comfort × bmic |  |  |  | 2 | -0.10 | 0.012 | -8.35 (6720) | <.0001 | -0.13 | - | -0.079 |
| Comfort × bmic |  |  |  | 25 | -0.07 | 0.012 | -5.30 (6719) | <.0001 | -0.089 | - | -0.041 |
| Comfort × bmic |  |  |  | 50 | -0.057 | 0.012 | -4.61 (6719) | <.0001 | -0.081 | - | -0.033 |
| Comfort × bmic |  |  |  | 75 | -0.046 | 0.012 | -3.72 (6719) | 0.0002 | -0.070 | - | -0.022 |
| Comfort × bmic |  |  |  | 91 | -0.028 | 0.012 | -2.24 (6719) | 0.025 | -0.052 | - | -0.003 |
| Comfort × bmic |  |  |  | 98 | -0.015 | 0.012 | -1.21 (6719) | 0.2281 | -0.039 | - | 0.009 |
| Comfort × bmic |  |  |  | 99.6 | 0.00 | . | . | . | . |  | . |
| Control |  |  |  |  | 0.062 | 0.025 | 2.48 (1176) | 0.0132 | 0.013 | - | 0.11 |
| Control × bmic |  |  |  | 2 | 0.00 | 0.025 | 0.16 (6719) | 0.8705 | -0.045 | - | 0.054 |
| Control × bmic |  |  |  | 25 | -0.012 | 0.025 | -0.48 (6719) | 0.6296 | -0.062 | - | 0.037 |
| Control × bmic |  |  |  | 50 | -0.013 | 0.025 | -0.51 (6719) | 0.6135 | -0.062 | - | 0.037 |
| Control × bmic |  |  |  | 75 | -0.0080 | 0.025 | -0.32 (6719) | 0.7518 | -0.057 | - | 0.041 |
| Control × bmic |  |  |  | 91 | -0.0023 | 0.025 | -0.090 (6719) | 0.9268 | -0.052 | - | 0.047 |
| Control × bmic |  |  |  | 98 | -0.0042 | 0.025 | -0.17 (6719) | 0.8682 | -0.054 | - | 0.045 |
| Control × bmic |  |  |  | 99.6 | 0.00 | . | . | . | . |  | . |

**1.3 Generalized linear mixed effect model (GLMM) of categorization accuracy**

For the categorical data, dichotomous match/mismatch was regressed upon a series of pre-specified predictors. As fixed effects, we tested: stimulus weight category (i.e., Wgt_cat: 1-4), participant group (i.e., Grp: HCPs and parents - PAR), and stimulus type (i.e., Image: younger girls GY, older girls GO, younger boys BY, and older boys BO). In addition, we tested the same covariates as for the VAS data analysis, above.

Table S5 shows a cross-tabulation of the LS-mean probability of a correct response, as a function of Image, Wgt-Cat, and Grp. These values are derived from the model parameters shown in Table S6. The significant fixed effect of weight category is illustrated by Table S5. Broadly the probability of a correct classification reduces systematically from level 2 to 4 for boys and girls. For images of boys, the probability of a correct response is lower for level 1 than 2, but slightly higher for level 1 than 2 for images of girls. The significant fixed effect of stimulus category is due to a lower probability of a correct categorization for boys (younger p = 0.30, older p=0.37) and a higher probability for girls (younger p = 0.44, older p=0.50). The significant fixed effect of participant group is due to a higher probability of correct weight classification by HCPs (p = 0.46) compared to parents (p = 0.36). The significant two-way interactions between weight category × stimulus category derives from the lower probability of correct responses to weight category 1 in boys (younger p = 0.69, older p = 0.56 ) than girls (younger p = 0.76, older p = 0.83 ), as well as lower probability responses to weight category 3 in boys (younger p = 0.18, older p = 0.32 ) than girls (younger p = 0.41, older p = 0.69 ). The significant two-way weight category × participant group derives primarily from the lower probability of correct responses to weight category 3 by parents (p = 0.32) than HCPs (p = 0.45). Finally, the significant two-way interaction weight category × Comfort is derived from the fact that increasing comfort scores led to increasingly correct responses for weight categories 3 and 4, but not categories 1 and 2.

Table S5 Tables of LS-Mean probabilities of a correct categorization from the GLMM model

|  |  | HCP |  |  |  |  | PARENTS |  |  |  |
| --- | --- | --- | --- | --- | --- | --- | --- | --- | --- | --- |
| Image | Wgt Cat | Probability | 95% CI | | |  | Probability | 95% CI | | |
| BO | 1 | 0.60 | 0.52 | - | 0.69 |  | 0.52 | 0.43 | - | 0.62 |
| BO | 2 | 0.93 | 0.90 | - | 0.95 |  | 0.93 | 0.90 | - | 0.95 |
| BO | 3 | 0.38 | 0.30 | - | 0.47 |  | 0.28 | 0.20 | - | 0.38 |
| BO | 4 | 0.05 | 0.03 | - | 0.08 |  | 0.02 | 0.01 | - | 0.05 |
|  | mean | 0.49 |  |  |  |  | 0.44 |  |  |  |
|  |  |  |  |  |  |  |  |  |  |  |
| BY | 1 | 0.73 | 0.64 | - | 0.80 |  | 0.67 | 0.58 | - | 0.75 |
| BY | 2 | 0.88 | 0.84 | - | 0.91 |  | 0.89 | 0.86 | - | 0.92 |
| BY | 3 | 0.22 | 0.16 | - | 0.30 |  | 0.13 | 0.08 | - | 0.20 |
| BY | 4 | 0.04 | 0.03 | - | 0.08 |  | 0.01 | 0.00 | - | 0.03 |
|  | mean | 0.47 |  |  |  |  | 0.43 |  |  |  |
|  |  |  |  |  |  |  |  |  |  |  |
| GO | 1 | 0.82 | 0.75 | - | 0.88 |  | 0.83 | 0.75 | - | 0.89 |
| GO | 2 | 0.77 | 0.73 | - | 0.81 |  | 0.82 | 0.77 | - | 0.86 |
| GO | 3 | 0.75 | 0.67 | - | 0.82 |  | 0.62 | 0.53 | - | 0.71 |
| GO | 4 | 0.06 | 0.04 | - | 0.10 |  | 0.04 | 0.02 | - | 0.07 |
|  | mean | 0.60 |  |  |  |  | 0.58 |  |  |  |
|  |  |  |  |  |  |  |  |  |  |  |
| GY | 1 | 0.76 | 0.68 | - | 0.83 |  | 0.75 | 0.67 | - | 0.82 |
| GY | 2 | 0.74 | 0.69 | - | 0.78 |  | 0.82 | 0.78 | - | 0.86 |
| GY | 3 | 0.47 | 0.38 | - | 0.56 |  | 0.37 | 0.29 | - | 0.47 |
| GY | 4 | 0.13 | 0.10 | - | 0.18 |  | 0.04 | 0.02 | - | 0.07 |
|  | mean | 0.53 |  |  |  |  | 0.50 |  |  |  |

The format and interpretation of Table S6 is the same as that for Tables S2 and S6. The only difference is that the estimates are expressed as logits. A logit is the natural log of an odd, where odd = p / (1-p). To illustrate, for the probabilities 0.2, 0.5, and 0.8, their respective logit transforms are: -1.386, 0.000, and 1.386, respectively.

Table S6 GLIMMIX parameters from categorical data

| Effect | Image | Grp | Wgt_cat | Estimate | SE | t value | p value | 95% CI | | |
| --- | --- | --- | --- | --- | --- | --- | --- | --- | --- | --- |
| Intercept |  |  |  | -3.12 | 0.53 | -5.85 (391) | <.0001 | -4.17 | - | -2.07 |
| Wgt_cat |  |  | 1 | 4.96 | 0.68 | 7.25 (6553) | <.0001 | 3.62 | - | 6.30 |
| Wgt_cat |  |  | 2 | 3.93 | 0.60 | 6.58 (6553) | <.0001 | 2.76 | - | 5.10 |
| Wgt_cat |  |  | 3 | 2.44 | 0.66 | 3.68 (6553) | 0.0002 | 1.14 | - | 3.74 |
| Wgt_cat |  |  | 4 | 0.0 | . | . | . | . |  | . |
|  | BO |  |  | -1.025 | 0.27 | -3.77 (6553) | 0.0002 | -1.56 | - | -0.49 |
| Image | BY |  |  | -1.33 | 0.30 | -4.42 (6553) | <.0001 | -1.92 | - | -0.74 |
| Image | GO |  |  | -0.58 | 0.24 | -2.40 (6553) | 0.02 | -1.057 | - | -0.11 |
| Image | GY |  |  | 0.0 | . | . | . | . |  | . |
| Grp |  | HCP |  | 1.11 | 0.22 | 5.040 (391) | <.0001 | 0.68 | - | 1.55 |
| Grp |  | PAR |  | 0.0 | . | . | . | . |  | . |
| Wgt_cat × Image | BO |  | 1 | 0.13 | 0.34 | 0.39 (6553) | 0.7 | -0.53 | - | 0.79 |
| Wgt_cat × Image | BY |  | 1 | 1.022 | 0.36 | 2.83 (6553) | 0.005 | 0.31 | - | 1.73 |
| Wgt_cat × Image | GO |  | 1 | 1.016 | 0.33 | 3.080 (6553) | 0.002 | 0.37 | - | 1.66 |
| Wgt_cat × Image | GY |  | 1 | 0.0 | . | . | . | . |  | . |
| Wgt_cat × Image | BO |  | 2 | 2.35 | 0.32 | 7.39 (6553) | <.0001 | 1.73 | - | 2.98 |
| Wgt_cat × Image | BY |  | 2 | 2.10 | 0.33 | 6.31 (6553) | <.0001 | 1.45 | - | 2.75 |
| Wgt_cat × Image | GO |  | 2 | 0.70 | 0.27 | 2.56 (6553) | 0.01 | 0.16 | - | 1.24 |
| Wgt_cat × Image | GY |  | 2 | 0.0 | . | . | . | . |  | . |
| Wgt_cat × Image | BO |  | 3 | 0.63 | 0.33 | 1.89 (6553) | 0.06 | -0.022 | - | 1.28 |
| Wgt_cat × Image | BY |  | 3 | 0.072 | 0.37 | 0.20 (6553) | 0.8 | -0.65 | - | 0.79 |
| Wgt_cat × Image | GO |  | 3 | 1.73 | 0.31 | 5.58 (6553) | <.0001 | 1.12 | - | 2.33 |
| Wgt_cat × Image | GY |  | 3 | 0.0 | . | . | . | . |  | . |
| Wgt_cat × Image | BO |  | 4 | 0.0 | . | . | . | . |  | . |
| Wgt_cat × Image | BY |  | 4 | 0.0 | . | . | . | . |  | . |
| Wgt_cat × Image | GO |  | 4 | 0.0 | . | . | . | . |  | . |
| Wgt_cat × Image | GY |  | 4 | 0.0 | . | . | . | . |  | . |
| Wgt_cat × Grp |  | HCP | 1 | -0.89 | 0.26 | -3.40 (6553) | 0.0007 | -1.41 | - | -0.38 |
| Wgt_cat × Grp |  | PAR | 1 | 0.00 | . | . | . | . |  | . |
| Wgt_cat × Grp |  | HCP | 2 | -1.36 | 0.24 | -5.61 (6553) | <.0001 | -1.84 | - | -0.88 |
| Wgt_cat × Grp |  | PAR | 2 | 0.00 | . | . | . | . |  | . |
| Wgt_cat × Grp |  | HCP | 3 | -0.56 | 0.26 | -2.12 (6553) | 0.03 | -1.071 | - | -0.042 |
| Wgt_cat × Grp |  | PAR | 3 | 0.0 | . | . | . | . |  | . |
| Wgt_cat × Grp |  | HCP | 4 | 0.0 | . | . | . | . |  | . |
| Wgt_cat × Grp |  | PAR | 4 | 0.0 | . | . | . | . |  | . |
| Comfort |  |  |  | 0.025 | 0.0039 | 6.42 (6553) | <.0001 | 0.018 | - | 0.033 |
| Comfort × Wgt_cat |  |  | 1 | -0.025 | 0.0046 | -5.32 (6553) | <.0001 | -0.034 | - | -0.015 |
| Comfort × Wgt_cat |  |  | 2 | -0.028 | 0.0043 | -6.48 (6553) | <.0001 | -0.036 | - | -0.019 |
| Comfort × Wgt_cat |  |  | 3 | -0.018 | 0.0046 | -3.86 (6553) | 0.0001 | -0.027 | - | -0.0088 |
| Comfort × Wgt_cat |  |  | 4 | 0.0 | . | . | . | . |  | . |
| Control |  |  |  | -0.016 | 0.01 | -2.53 (6553) | 0.01 | -0.028 | - | -0.004 |
| Control × Wgt_cat |  |  | 1 | 0.005 | 0.01 | 0.6 (6553) | 0.5 | -0.011 | - | 0.021 |
| Control × Wgt_cat |  |  | 2 | 0.025 | 0.01 | 3.51 (6553) | 0.0004 | 0.011 | - | 0.039 |
| Control × Wgt_cat |  |  | 3 | 0.012 | 0.01 | 1.48 (6553) | 0.1 | -0.0038 | - | 0.027 |
| Control × Wgt_cat |  |  | 4 | 0.0 | . | . | . | . |  | . |

**2. Sensitivity analysis**

While all parents had children under the age of 18, by definition, it proved hard to find HCPs who did not have children under the age of 18. Indeed, 89 HCPs self-reported having children. This meant that, to some extent, participant group status (i.e., parent versus HCP) was confounded with parenthood per se. In all our analyses (i.e., normalized VAS, non-normalized VAS, and weight categorization data), if we included a factor (referred to as CHU18) for whether or not a given participant had a child/children under 18 or not, this factor never made a statistically significant contribution to the models. To explore this issue further, we carried out a sensitivity analysis, which is reported below.

We used a simulation method to randomly convert 20/89, 40/89, 60/89, 80/89, and 88/89 HCPs to non-parental status. For each level of “knockout”, we carried out 1000 resamplings, computed the appropriate LME / GLMM model separately for the normalized VAS, non-normalized VAS, and categorical data, and recorded the Akaike Information Criterion (AICc) as an index of model fit; better fitting models have smaller AICc values. For every resample, we then calculated the difference in AICc between the original models as reported in Tables S2, S4, and S6, and the knockout models. In a final step we calculated the mean and standard error for these differences in AICc as a function of knockout level. Figure S1 shows plots of AICc difference as a function of knockout level, separately for: a) the normalized VAS data, b) the non-normalized VAS data, and c) the categorization data. To compare model fits (in this case base model with knockout models), we adopted the criteria of Burnham and Anderson (2002): model comparisons where Δ AICci ≤ 2 have no support (evidence) for retaining a new variable, those in which 4 ≤ Δ AICci ≤ 7 have considerable support, and models having Δ AICci > 10 have substantial support. With the exception of the normalized VAS data, where only 1/89 participants retain parental status, the changes in AICc as a result of reducing the proportion of parents would not be considered as good model improvements. Therefore, in the light of these results, together with the lack of a significant impact of CHU18 on the original models, we argue that any confounding of group status (i.e., parent versus HCP) with parenthood, was negligible.


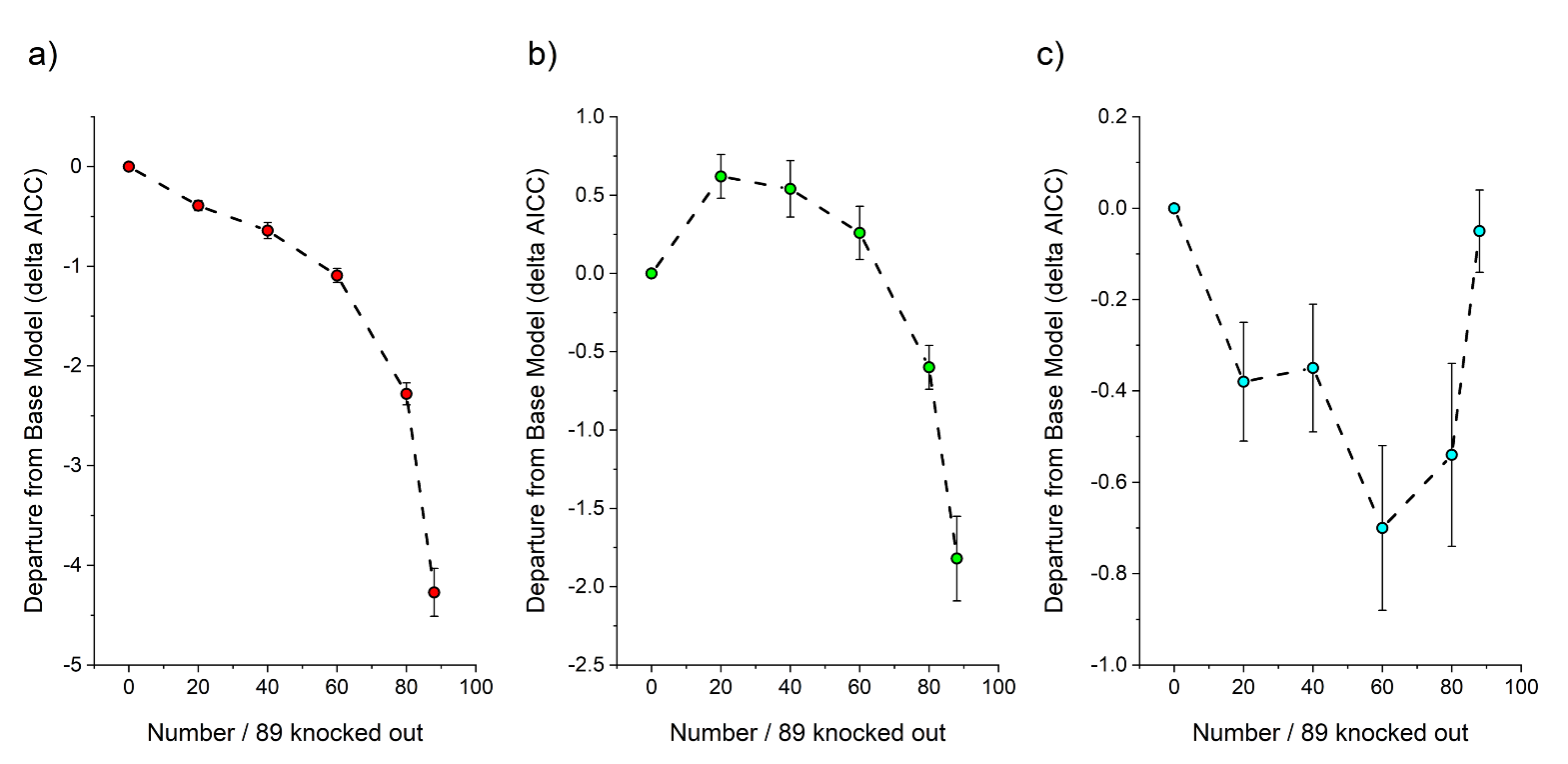


Figure S1. Plots of delta AICc as a function of the number of HCPs knocked out of parenthood status for: a) the normalized VAS data, b) the non-normalized VAS data, and c) the categorization data.

**3.** **Alternative explanation for variation in categorization accuracy?**

One reviewer suggested that one explanation for our findings could be a general tendency to score higher on the categorisation task by some participants. If so, this should lead to an increase in proportion of correct responses for the overweight/very-overweight child stimuli, but the upward shift in the responses should also lead to a decrease in the proportion of correct underweight responses (as underweight bodies will be more likely to be rated as healthy weight).

To address this plausible alternative, we carried out the following additional analysis. The GLMM reported in Tables S5 and S6 generates a predicted probability of a correct response for each trial. For simplicity, we collapsed the per trial data across weight categories 3 and 4 (i.e., over-weight and very over-weight) and henceforth refer to this as OW. We then plotted the predicted probabilities of correct responses to under-weight (UW) and healthy weight (HW) (both on the y-axis) as a function of the predicted probability of a correct response to OW (collapsed across stimulus sex, age, and participant group – x-axis) as shown in Figure S2. The respective correlations for this pattern of results are: UW v HW, r = -0.50, p < .0001; UW v OW, r = 0.36, p < .0001; HW v OW, r = -0.24, P < .0001. The results are plotted in Figure S2, which shows clearly that as the probability of correctly categorising overweight bodies increases, so does the probability of correctly categorising underweight bodies. This is the opposite of what would be predicted by the reviewer’s suggestion.


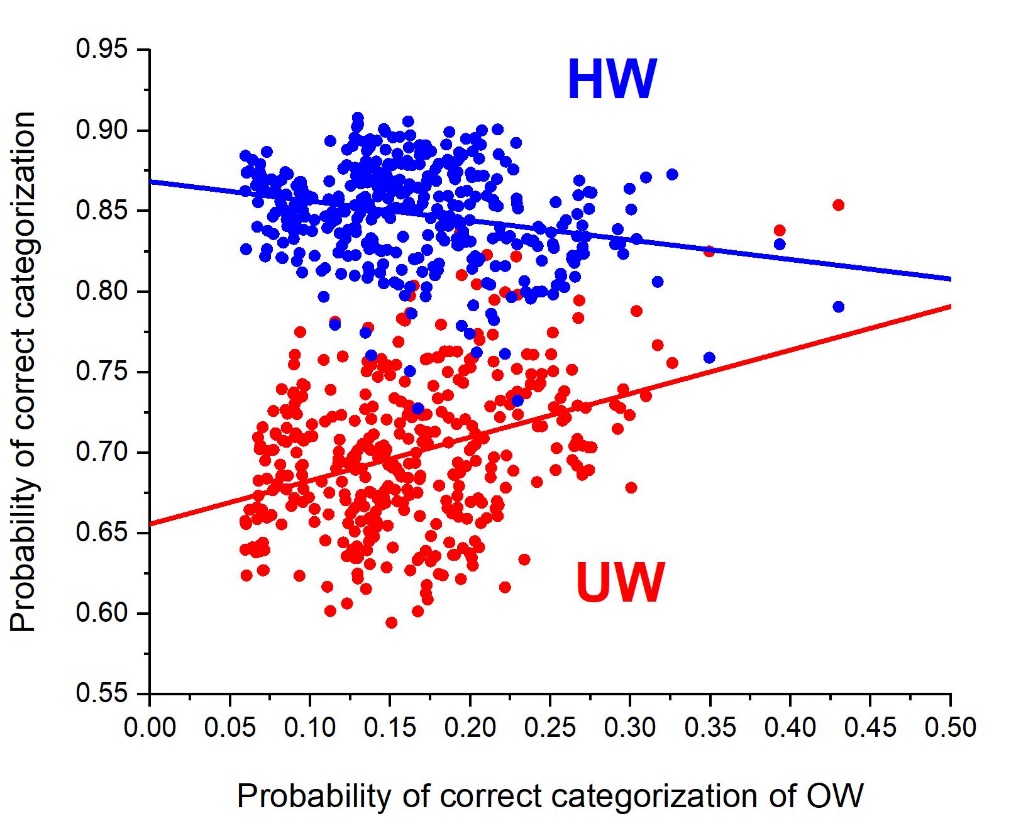


Figure S2. Plot of the predicted probability of correct weight categorization for UW and HW images plotted as a function of the probability of correct categorization of OW images.

Instead, we suggest an interpretation framed in terms of specificity. Participants who make very vague, non-specific responses are likely to capture most of the mid-range of stimulus weights and classify them correctly, by default. However, for such individuals, this would also mean that the boundaries for the extreme underweight and overweight classifications are compressed into narrower ranges than normal, hence leading to less accurate responses for these stimuli. So, poor specificity should lead to higher correct response rates for healthy weight and lower correct response rates for underweight and overweight stimuli. Conversely, participants who try to be more specific in their responses will narrow the body size range for their healthy weight categorizations, and widen the body size ranges for their underweight and overweight categorizations. Consequently, this will decrease the correct rate for healthy weight, but increase it for the underweight and overweight categories, leading to the patterns of correlation above.
